# One-month recovery profile and prevalence and predictors of quality of recovery after painful day case surgery: Secondary analysis of a randomized controlled trial

**DOI:** 10.1371/journal.pone.0245774

**Published:** 2021-01-26

**Authors:** Björn Stessel, Maarten Hendrickx, Caroline Pelckmans, Gerrit De Wachter, Bart Appeltans, Geert Braeken, Jeroen Herbots, Elbert Joosten, Marc Van de Velde, Wolfgang F. F. A. Buhre

**Affiliations:** 1 Department of Anesthesiology and Pain Medicine, Jessa Hospital, Hasselt, Belgium; 2 Department of Anesthesiology and Pain Management, Maastricht University Medical Center+, Maastricht, The Netherlands; 3 UHasselt, Faculty of Medicine and Life Sciences, LCRC, Agoralaan, Diepenbeek, Belgium; 4 Department of Anaesthesiology and Pain Medicine, University Hospital, Leuven, Belgium; 5 Department of Orthopedic Surgery, Jessa Hospital, Hasselt, Belgium; 6 Department of Abdominal Surgery, Jessa Hospital, Hasselt, Belgium; 7 Department of Clinical Epidemiology and Medical Technology Assessment, Maastricht University Medical Center+, Maastricht, The Netherlands; 8 School for Mental Health and Neuroscience (MHeNS), Faculty of Health, Medicine and Life Sciences, Maastricht University, Maastricht, The Netherlands; University of Texas Medical Branch at Galveston, UNITED STATES

## Abstract

**Background/Objectives:**

This study aimed to study one-month recovery profile and to identify predictors of Quality of Recovery (QOR) after painful day surgery and investigate the influence of pain therapy on QOR.

**Methods/Design:**

This is a secondary analysis of a single-centre, randomised controlled trial of 200 patients undergoing ambulatory haemorrhoid surgery, arthroscopic shoulder or knee surgery, or inguinal hernia repair between January 2016 and March 2017. Primary endpoints were one-month recovery profile and prevalence of poor/good QOR measured by the Functional Recovery Index (FRI), the Global Surgical Recovery index and the EuroQol questionnaire at postoperative day (POD) 1 to 4, 7, 14 and 28. Multiple logistic regression analysis was performed to determine predictors of QOR at POD 7, 14, and 28. Differences in QOR between pain treatment groups were analysed using the Mann-Whitney U test.

**Results:**

Four weeks after haemorrhoid surgery, inguinal hernia repair, arthroscopic knee and arthroscopic shoulder surgery, good QOR was present in 71%, 76%, 57% and 24% respectively. Poor QOR was present in 5%, 0%, 7% and 29%, respectively. At POD 7 and POD 28, predictors for poor/intermediate QOR were type of surgery and a high postoperative pain level at POD 4. Male gender was another predictor at POD 7. Female gender and having a paid job were also predictors at POD 28. Type of surgery and long term fear of surgery were predictors at POD 14. No significant differences in total FRI scores were found between the two different pain treatment groups.

**Conclusions:**

The present study shows a procedure-specific variation in recovery profile in the 4-week period after painful day surgery. The best predictors for short-term (POD 7) and long-term (POD 28) poor/intermediate QOR were a high postoperative pain level at POD 4 and type of surgery. Different pain treatment regimens did not result in differences in recovery profile.

**Trial registration:**

European Union Clinical Trials Register 2015-003987-35.

## Background

Recent developments in assessment of quality parameters after surgery have led to the implementation of Quality of Recovery (QOR) as a principal endpoint after day case surgery. The QOR is related to various aspects of patients´ daily living after discharge at home [1]. QOR is a complex phenomenon where many aspects in physical, psychological and social health are involved and which is a subjective experience by the patient [1]. Thus, the key for evaluating QOR is an assessment from the patient´s perspective by using the patient own ratings.

After day case surgery, both patients and health care providers expect a good quality and fast recovery including a rapid return to daily and work activities without suffering from moderate to severe pain or functional disability [2, 3]. However, the recovery process is quite variable and after some types of surgery, complete recovery may take several weeks and even months [3, 4]. Furthermore, most of the recovery process occurs at home and places greater responsibility on the patients and their relatives [5]. The recovery process relies on well-documented oral and written information to evaluate if their perceived recovery trajectory is normal or pathological. It has been shown that better patient information can reduce perioperative anxiety itself and the elicited negative effects on outcome [6]. Also, inadequate functional health literacy, defined as a patient´s capacity to gain and process information to maintain good health, seems to be associated with poorer QOR after day case surgery [5]. Consequently, excellent information provision seems crucial to promote a good QOR. To provide this information, recovery profile, i.e. the course of recovery at different time points after different types of day case surgery should be mapped.

Some patient- and surgery-related characteristics may be associated with an unfavourable outcome [4, 7]. As a result, there is a need for more in-depth understanding of the variability of QOR in the first month after day case surgery and the influence of demographic, psychological, social and perioperative factors on global perceived QOR. This knowledge would allow to discriminate between a normal and pathological recovery trajectory. Furthermore, identification of predictors of poor QOR in the first month after day case surgery may lead to the introduction of techniques aiming at improving QOR. Finally, the urge for unplanned contacts with health professionals may be reduced, patient expectations may be adjusted and follow-up care can be scheduled [8–10].

It has already been suggested that adequate acute postoperative pain management may result in an improvement of the QOR in the post discharge period [11, 12]. Consequently, the influence and effect of pain therapy on the recovery profile might be an important starting point to improve QOR after surgery in the future.

There are several patient-reported instruments for assessing QOR after day case surgery [13, 14]. The convenient and validated Global Surgical Recovery (GSR) index represents a single question about the extent to which patients considered themselves to be globally recovered from the surgery [15]. The functional recovery index (FRI) is a tool specifically developed to assess different domains of recovery after day case surgery [1, 16]. Also, Quality Of Life (QOL) questionnaires may fulfil the requirements as useful indicators of surgical recovery [4]. However, so far there is no general agreement on the optimal instruments for evaluating recovery and outcome following day case surgery [17].

This study aimed to analyse the QOR at different time points after four specific types of day case surgery, each known to have a high incidence of poor QOR at the fourth postoperative day (POD) [18]. We hypothesized that each type of day case surgery included in our trial will have a unique recovery profile. In addition, we aimed to identify predictors of poor/intermediate QOR after day case surgery and to investigate the influence of acute pain and pain therapy on recovery profile.

## Materials and methods

The present prospective, observational study analysed data from a large randomized trial investigating if a combination of metamizole and paracetamol is non-inferior to a combination of ibuprofen and paracetamol in the treatment of acute postoperative pain at home after painful day case surgery executed in the JESSA Hospital Hasselt, Belgium. These data have been published in the *European Journal of Anaesthesiology* [19]. The complete study protocol has also been published in *Trials* [20]. Briefly, this study was approved by the ethical committee of the JESSA Hospital Hasselt, Belgium (Chairperson Dr. Koen Magerman, registration number 15.105/pijn15.02) on 21 September 2015 and by the European Union Drug Regulating Authorities Clinical Trials (EudraCT Number 2015-003987-35).

After obtaining written informed consent, we recruited 200 patients scheduled for elective day case haemorrhoid surgery (n = 50), arthroscopic shoulder (n = 50) or knee (n = 50) surgery, or inguinal hernia repair (n = 50) between 28 January 2016 and 31 March 2017. Patients with ASA physical status 1–3 between 18 and 70 years of age and a Body Weight > 50kg were included. Exclusion criteria were inpatient surgery, pregnancy, cognitive impairment, no understanding of the Dutch language, preoperative pharmacologic pain treatment and/or a history of chronic pain, a history of substance abuse, or use of medication with a suppressive effect on the central nervous system, allergy to or a contraindication for taking the study medication (e.g. paracetamol, metamizole, ibuprofen or another NSAID), fever or other signs of infection and for patients undergoing arthroscopy shoulder: refusal of an interscalene block. Baseline assessment measurements included the participants’ age, gender, Body Mass Index (BMI), ASA classification, work status, highest level of education, pre-operative pain intensity (on a Numerical Rating Scale (NRS) with 0 indicating no pain and 10 indicating worst pain) and expected postoperative pain intensity (NRS-score). The eight-item Surgical Fear Questionnaire (SFQ) was included to assess baseline fear of the surgical procedure [18, 21]. Within the SFQ, four items refer to fear of short term consequences (e.g. pain, side effects) and four items refer to fear of long term consequences (e.g. deterioration of health) [21].

### Perioperative procedure

All patients scheduled for an arthroscopic shoulder procedure received an interscalene block preoperatively. In accordance with local practice, general anaesthesia was induced with alfentanil 10 mcg/kg i.v., sufentanil 0.15 mcg/kg i.v. and propofol 2mg/kg i.v.. Patients undergoing arthroscopic shoulder surgery or laparoscopic inguinal hernia repair also received rocuronium 0.6 mg/kg i.v. before endotracheal intubation. A laryngeal mask airway was inserted in all other patients. Anaesthesia was maintained with sevoflurane in a mixture of 50:50 air/oxygen. All patients received ondansetron 4 mg IV at the end of surgery. Duration of surgery was recorded.

Postoperatively, all patients were treated with subsequent bolus injections of piritramide 2mg intravenously until an NRS ≤ 3 was achieved in the Post Anaesthesia Care Unit (PACU). Before hospital discharge, patients received the study medication (metamizole + paracetamol versus ibuprofen + paracetamol) and instructions for use.

### Outcome measures

The primary outcome measure is the recovery profile measured by 3 different tools at baseline and by telephone call at POD 1 to 4, 7, 14 and 28 to assess the course of recovery over time. First, the FRI is a validated questionnaire specifically developed to assess post-discharge functional QOR after day case surgery and covers 14 items divided in to 3 different domains: pain and social activity consisting of 7 questions involving work, physical activity and pain; lower limb activity involving 4 questions regarding different movements of the legs; and general physical activity involving 3 questions regarding dressing, washing, and resting. All these items are scored on a scale from 0 to 10, with 0 = no difficulty and 10 = extreme difficulty with the activity [1]. Second, the validated GSR index represents a single question about the extent to which patients considered themselves to be recovered from the surgery with 0% indicating not recovered at all and 100% indicating a full recovery [15]. Third, the 5-dimensional EuroQol (EQ-5D) questionnaire is a widely used non-disease specific instrument developed for describing and valuing health-related QOL [22] and has already been used to assess intermediate (4 days) and late (2 weeks– 6 months) QOR after day case surgery [18, 23, 24]. The EQ5D focuses on five topics i.e. mobility, self-care, daily activities, pain/discomfort and anxiety [22]. These are ranged from 1 indicating no difficulty at all to 5 indicating extremely difficult/being incapable [22]. In addition, we assessed the prevalence of poor, intermediate and good QOR and tried to identify predictors of poor QOR, based on the following definition: QOR is defined as good if both the GSR index is >80% [25, 26] and if the postoperative EQ-5D is unchanged or improved as compared with baseline [18]. QOR is defined as poor if both the GSR index is ≤80% and if the postoperative EQ-5D is reduced as compared with baseline [18]. QOR is defined as intermediate in all other cases [18]. Furthermore, we also applied an alternative definition of recovery, based on the FRI score: recovery was predefined into recovered and improved [3]. *Recovered* was defined as the absence of a significant difference between total median postoperative FRI score and baseline FRI score. *Improved* was defined as a significant improvement of total median postoperative FRI score compared with baseline. To assess the recovery process of individual patients, an FRI score between 5 points higher and 10 points higher than baseline FRI score was considered recovered. An FRI score > 10 points lower than baseline was considered improved. Finally, we assessed potential differences in QOR between the 2 different pain treatment groups.

Secondary outcome measures included pre-operative and post-operative pain intensity and were evaluated at baseline and POD 1 to 4, 7, 14 and 28 with a Numerical Rating Scale (NRS) with 0 indicating no pain and 10 indicating worst pain.

### Statistical analysis

All baseline characteristics and the primary outcome measure are presented as median (25th - 75th percentile) or as absolute numbers (%). Missing baseline values were imputed using multiple imputation. The number of imputations was set to 10. The Wilcoxon signed-rank test and a Bonferroni adjustment were used to compare median postoperative FRI scores with baseline. Differences in QOR between pain treatment groups were analysed using the Mann-Whitney U test.

For the prediction of poor and intermediate versus good QOR at respectively POD 7, 14 and 28 postoperatively, a multiple logistic regression analysis was performed. The following variables were tested for their predictive value: age, gender, baseline total EQ-5D, education, profession, BMI, type of surgery, fear of short- and long-term aspects of surgery, pre-operative pain intensity, duration of surgery, type of surgery and pain intensity at POD 4. The goodness of fit (GOF) of the logistic model was evaluated with the Hosmer and Lemeshow test and the quality of prediction was evaluated with the Nagelkerke R square test. Based on the classification table, we calculated the False Positive Rate. A p-value <0.05 is considered statistically significant. All analyses were performed using SPSS 24.0 (IBM® SPSS® Inc, Chicago, Illinois, USA). Graphs were made using Prism 7.0 (Prism®, GraphPad Software, Inc, La Jolla, California, USA).

## Results

**Fig 1:**
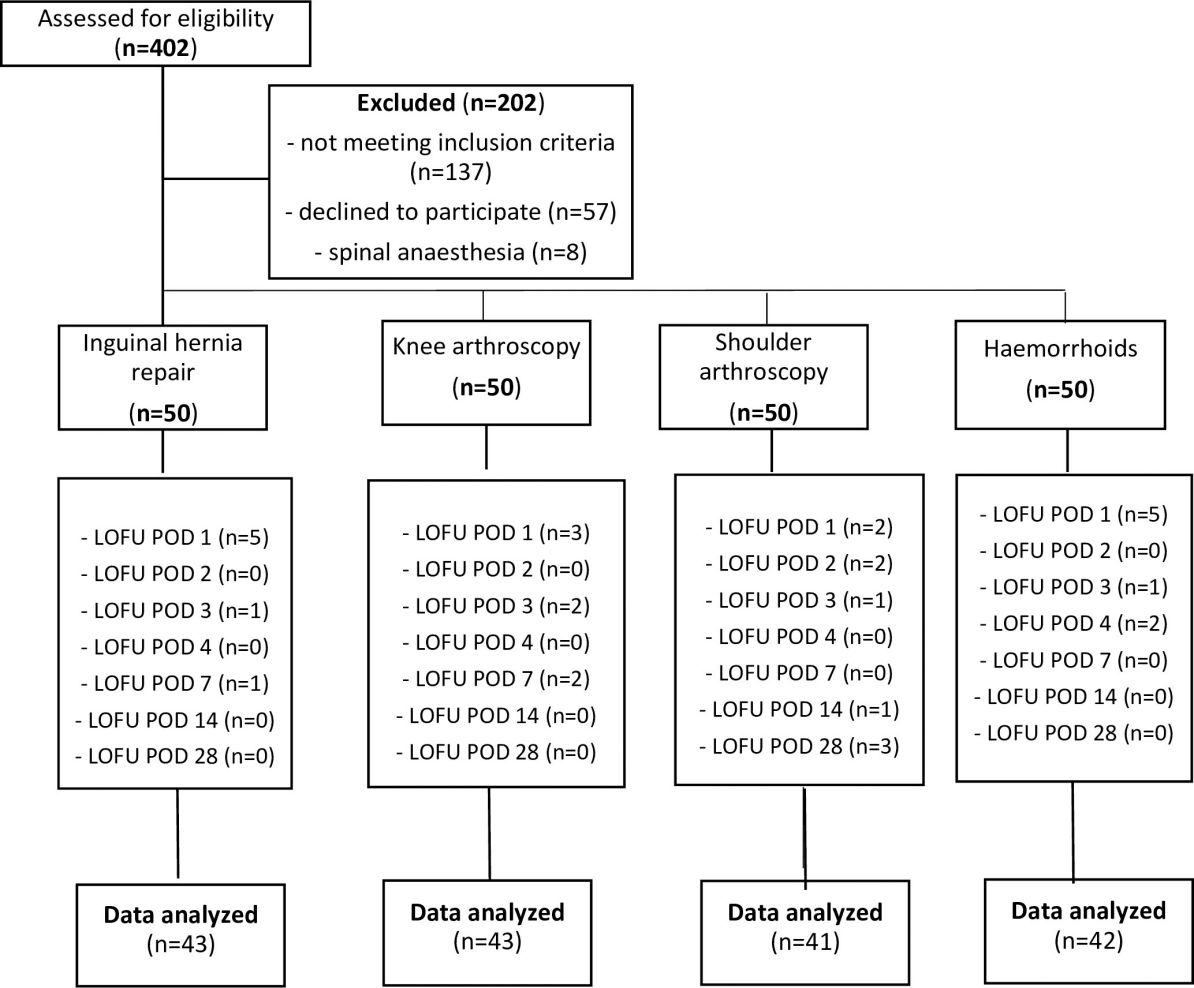
Study flow diagram. LOFU: Loss to follow-up, POD: post-operative day.

A flow chart of patient selection and exclusion is presented in Fig 1. Four hundred and two patients were screened for eligibility, of which 202 patients were excluded due to refusal to participate (n = 57), not meeting the inclusion criteria (n = 137) or undergoing spinal anaesthesia (n = 8). This resulted in data of 169 patients for the final analysis. No adverse events were reported during the study. Baseline and perioperative characteristics of all included patients are presented in Table 1.

**Table 1:** Baseline characteristics.

	Haemorrhoid surgery (n = 42)	Inguinal hernia repair (n = 43)	Arthroscopy knee (n = 43)	Arthroscopy shoulder (n = 41)
Age (years)	50.0 (45.7–52.6)	58.0 (50.6–58.2)	50.0 (46.3–53.7)	50.0 (45.6–51.4)
BMI (kg/m^2^)	25.8 (24.9–27.3)	24.4 (24.1–26.0)	26.5 (24.1–26.0)	26.5 (25.8–28.2)
Gender (male/female)	25/17 (59.5/40.5)	40/3 (93.0/7.0)	33/10 (76.7/23.3)	17/24 (41.4/58.6)
Employment status				
Unemployed	11 (26.2)	21 (48.8)	12 (28.0)	12 (29.2)
Paid work	31 (73.8)	22 (51.2)	31 (72.0)	29 (70.8)
Missing data	0 (0.0)	0 (0.0)	0 (0.0)	0 (0.0)
Education level				
Primary‎/junior secondary education	9 (21.4)	10 (23.3)	9 (20.9)	13 (31.7)
Upper secondary education	23 (54.7)	18 (41.8)	23 (53.5)	12 (29.2)
Higher education	10 (23.9)	15 (34.9)	11 (25.6)	16 (34.3)
Missing data	0 (0.0)	0 (0.0)	0 (0.0)	0 (0.0)
ASA-classification				
ASA I	12 (28.5)	17 (39.5)	20 (46.5)	11 (26.8)
ASA II	26 (62.4)	24 (55.9)	15 (34.9)	23 (56.1)
ASA III	2 (4.7)	0 (0.0)	2 (4.6)	3 (7.3)
Missing data	2 (4.7)	2 (4.6)	6 (14.0)	4 (9.8)
Last week: pain associated with the condition (yes/no/missing)?	20/20/2 (47.6/47.6/4.8)	21/22/0 (48.8/51.2/0.0)	32/11/0 (74.4/25.6/0.0)	39/2/0 (95.1/4.9/0.0)
Average pain	4.0 (3.8–6.0)	3.0 (2.8–4.5)	4.5 (3.8–5.2)	6.0 (6.1–7.2)
Influence pain on daily activities	4.5 (3.3–5.9)	2.0 (1.9–3.9)	4 (3.3–5.0)	6.0 (5.5–6.8)
Short-term surgical fear (0–40)	15.0 (12.7–18.3)	10.5 (9.1–14.6)	9.0 (7.4–12.6)	13.0 (12.3–19.0)
Long-term surgical fear (0–40)	5.0 (5.5–10.0)	5.0 (4.4–9.1)	7.0 (6.1–10.0)	12.0 (9.6–15.0)
Expected pain (0–10)	7.0 (5.8–7.2)	5.0 (3.0–6.0)	5.0 (4.4–5.7)	5.0 (4.1–5.7)
Duration of surgery (min)	14.0 (13.0–18.1)	22.0 (22.0–29.4)	28.5 (27.5–37.6)	45.0 (42.3–51.0)

Data are presented as median (25^th^ - 75^th^ percentile) or as absolute numbers (%). BMI: body mass index, ASA: American Society of Anaesthesiologists.

**Fig 2:**
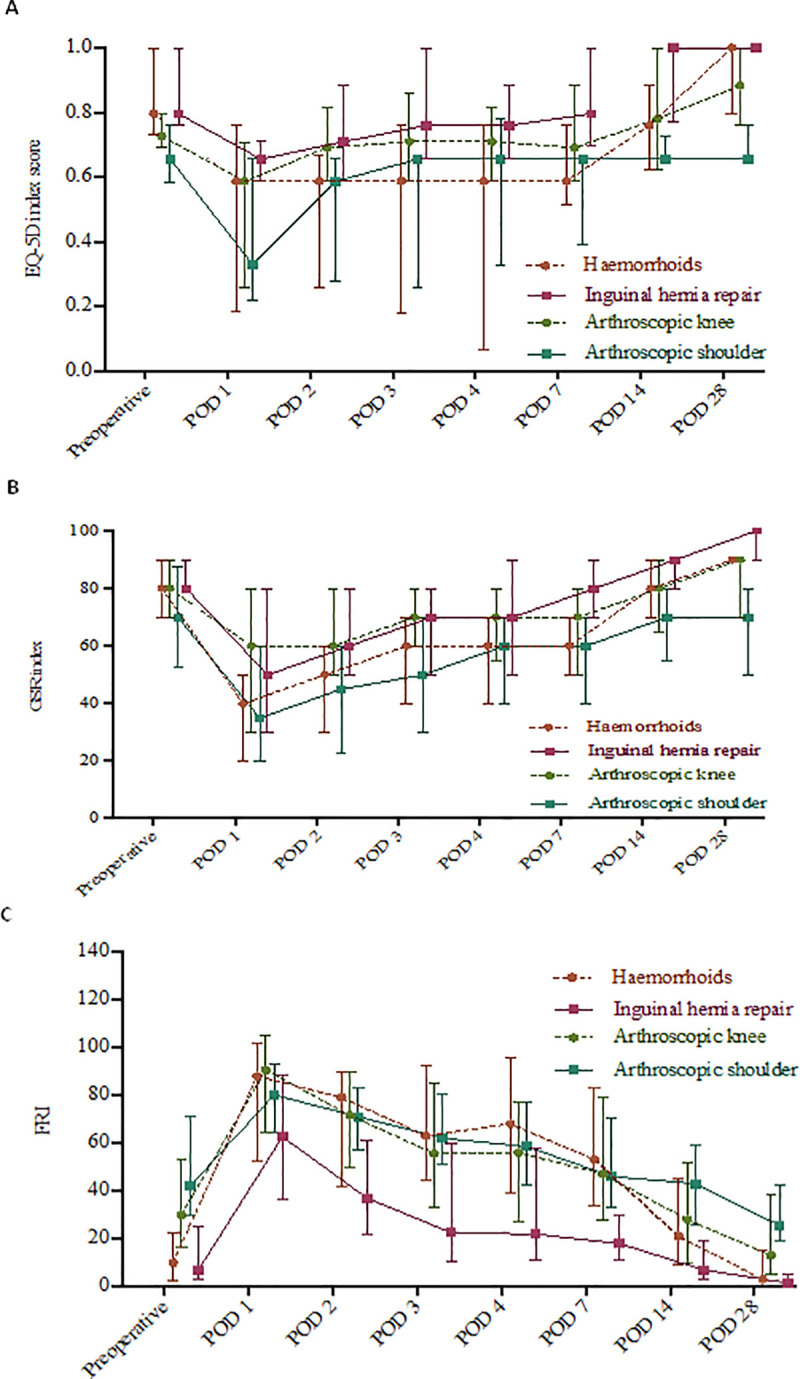
Median and interquartile range of EQ-5D index scores (A), GSR index scores (B) and FRI total scores (C).

Fig 2 shows the medians and interquartile ranges of EQ-5D index scores, GSR index scores and total FRI scores at baseline and at POD 1 to 4, 7, 14 and 28, respectively. In contrast to the EQ-5D and GSR-I, a high total FRI score indicated poor QOR and vice versa.

The prevalence of poor, intermediate and good QOR stratified per type of surgery is presented in Table 2. Four weeks after day case haemorrhoid surgery, inguinal hernia repair, arthroscopic knee and arthroscopic shoulder surgery, poor QOR was found in 5%, 0%, 7% and 29% of patients, respectively. Furthermore, good QOR was present in 71%, 76%, 57% and 24% of patients four weeks after the former four types of surgery, respectively.

**Table 2:** Number of patients (%) reporting a good, intermediate and poor recovery after ambulatory surgery on different postoperative days (POD).

	Good recovery	Intermediate recovery	Poor recovery
Haemorrhoid surgery (n = 42)			
POD 1	3 (7.14%)	5 (11.9%)	34 (80.95%)
POD 2	2 (4.76%)	8 (19.04%)	32 (76.19%)
POD 3	2 (4.76%)	7 (16.66%)	33 (78.57%)
POD 4	5 (11.9%)	7 (16.66%)	30 (71.43%)
POD 7	5 (11.9%)	6 (14.28%)	31 (73.81%)
POD 14	18 (42.86%)	13 (30.95%)	11 (26.19%)
POD 28	30 (71.43%)	10 (23.81%)	2 (4.76%)
Inguinal hernia repair (n = 43)			
POD 1	6 (14.95%)	14 (32.55%)	23 (52.50%)
POD 2	8 (18.60%)	17 (39.53%)	18 (41.87%)
POD 3	9 (20.93%)	20 (46.51%)	14 (32.56%)
POD 4	11 (25.57%)	18 (41.87%)	14 (32.56%)
POD 7	18 (41.87%)	18 (41.87%)	7 (16.26%)
POD 14	25 (58.14%)	14 (32.55%)	4 (9.31%)
POD 28	32 (74.42%)	11 (25.58%)	0 (0%)
Knee arthroscopy (n = 43)			
POD 1	3 (6.98%)	13 (30.23%)	27 (62.79%)
POD 2	7 (16.28%)	14 (32.55%)	22 (51.17%)
POD 3	9 (20.9.%)	18 (41.86%)	16 (37.54%)
POD 4	9 (20.9%)	18 (41.86%)	16 (37.54%)
POD 7	13 (30.23%)	15 (39.54%)	13 (30.23%)
POD 14	22 (48.84%)	13 (30.23%)	8 (20.93%)
POD 28	25 (58.14%)	15 (34.88%)	3 (6.98%)
Shoulder arthroscopy (n = 41)			
POD 1	5 (12.19%)	10 (24.39%)	26 (63.41%)
POD 2	5 (12.19%)	12 (29.27%)	24 (53.53%)
POD 3	1 (2.4%)	20 (48.78%)	20 (48.78%)
POD 4	6 (14.63%)	22 (53.66%)	13 (31.70%)
POD 7	3 (7.32%)	25 (60.97%)	13 (31.70%)
POD 14	12 (29.27%)	21 (51.22%)	8 (19.51%)
POD 28	10 (24.39%)	19 (46.34%)	12 (29.27%)

QOR is defined as good if both the GSR index is >80% and if the postoperative EQ-5D is unchanged or improved as compared with baseline. QOR is defined as poor if both the GSR index is ≤80% and if the postoperative EQ-5D is reduced as compared with baseline. QOR is defined as intermediate in all other cases [18].

Baseline total median FRI score was compared with total median FRI scores at follow-up POD 1 to 4, 7, 14 and 28, based on a Wilcoxon signed-rank test and Bonferroni adjustment (Table 3). Based on total median scores, recovery was complete two weeks after haemorrhoid surgery, arthroscopic knee surgery and inguinal hernia repair. Improvement was present four weeks after inguinal hernia repair and arthroscopic shoulder surgery.

**Table 3:** FRI baseline compared to follow-up days.

	Haemorrhoid surgery	Inguinal hernia repair	Arthroscopy knee	Arthroscopy shoulder
Baseline versus POD1	-61.0 (-87.0;-35.3)*	-49.0(-65.0;-17)*	-54.0(-70.0;-31.5)*	-27.0(-50.5;-10.0)*
Baseline versus POD2	-50.5(-76.0;-25.5)*	-24.0(-49.0;-6.5)*	-37.0(-57.0;-14.5)*	-18.0(-42.0;-3.0)*
Baseline versus POD3	-48.0(-67.8;-25.8)*	-13.0(-43.5;0.0)*	-24.5(-37.0;-0.3)	-8.5(-34.3;3.0)
Baseline versus POD4	-42.0(-70.0;-28.0)*	-15.0(-39.0;-1.0)	-20.0(-40.0;-2.0)	-7.0(-26.0;16.0)
Baseline versus POD7	-32.0(-58.3;-19.0)*	-8.0(-14.0;0.5)	-15.0(-32.0;3.8)	-3.0(-17.0;21.0)
Baseline versus POD14	-5.5(-19.3;0.5)	-1.0(-3.0;8.0)	-1.0(-10.8;26.3)	5.0(-13.0;26.0)
Baseline versus POD28	3.0(-0.8;16.0)	5.5(0.0;33.3)*	18.5(1.0;37.0)	18.0(2.5;37.0)*

Differences (baseline—postoperative day) are presented as median (25^th^; 75^th^ percentile).

^*^P-value <0.0018, (Wilcoxon signed rank test with Bonferroni adjustment). POD: PostOperative Day.

**Fig 3:**
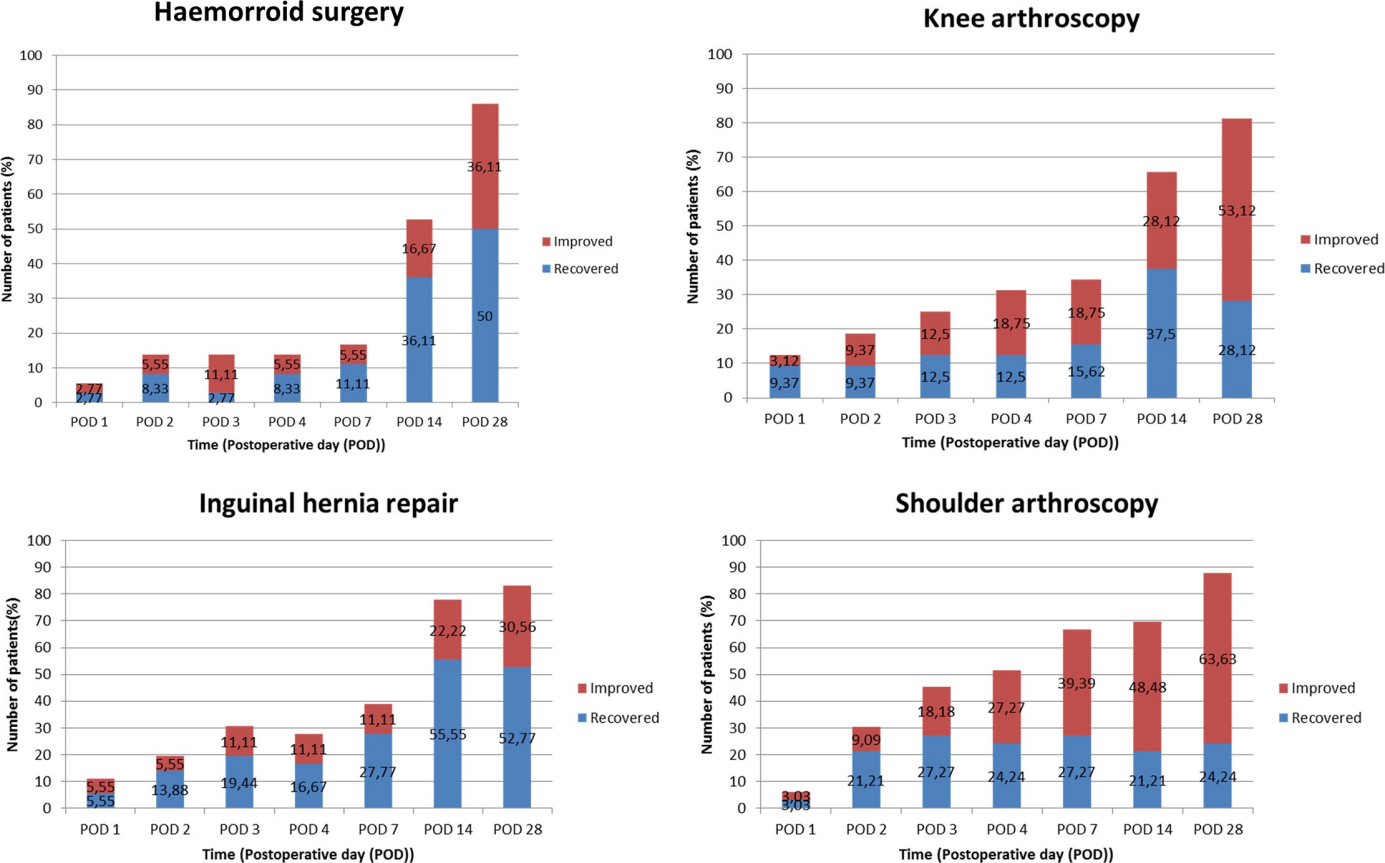
Number of patients recovered (A) and improved (B) at different time points compared to baseline FRI after ambulatory surgery. POD: postoperative day. An FRI score in the range of 5 points higher or 10 points lower than baseline FRI is considered recovered. An FRI score 10 points lower than baseline is considered improved.

The percentage of *recovered* and *improved* individual patients is presented in Fig 3. Four weeks after haemorrhoid surgery, arthroscopic knee surgery, inguinal hernia repair and arthroscopic shoulder surgery, recovery was incomplete in 14%, 17%, 19% and 13% of individual patients, respectively.

### Predictors and prediction model of poor/intermediate versus good QOR at POD 7, 14 and 28

The results of the multiple logistic regression analysis for poor/intermediate versus good QOR at POD 7, 14 and 28 are presented in Table 4. At POD 7, male gender, postoperative pain on POD4 and type of surgery were found to be the most important predictors for poor/intermediate QOR.

**Table 4:** Multiple logistic regression analysis for poor/intermediate versus good QOR at POD 7, 14 and 28.

Independent variable	Poor/Intermediate vs Good QOR Day 7	p-value	Poor/Intermediate vs Good QOR Day 14	p-value	Poor/Intermediate vs Good QOR Day 28	p-value
Gender	3.79 (1.11, 12.95)	**0.03**	0.86 (0.33, 2.22)	0.75	0.25 (0.10, 0.66)	**<0.01**
Profession	1.08 (0.36, 3.21)	0.89	1.58 (0.65, 3.83)	0.31	4.09 (1.55, 10.80)	**<0.01**
Education	1.40 (0.44, 4.44)	0.56	0.67 (0.29, 1.54)	0.34	0.85 (0.36, 2.02)	0.71
BMI	0.95 (0.83, 1.08)	0.43	1.07 (0.95, 1.19)	0.25	1.01 (0.90, 1.12)	0.93
Duration of surgery	0.99 (0.96, 1.03)	0.78	1.00 (0.97, 1.03)	0.99	1.01 (0.98, 1.04)	0.58
Fear short term	0.99 (0.92, 1.06)	0.78	0.97 (0.92, 1.02)	0.24	0.97 (0.92, 1.03)	0.37
Fear long term	1.01 (0.92, 1.11)	0.82	1.07 (1.00, 1.15)	**0.05**	1.04 (0.97, 1.11)	0.30
Preoperative pain	1.26 (0.38, 4.15)	0.70	1.20 (0.48, 3.05)	0.70	0.76 (0.28, 2.04)	0.59
Postoperative pain on day 4	4.82 (1.15, 20.21)	**0.03**	2.11 (0.81, 5.50)	0.13	3.46 (1.25, 9.56)	**0.02**
Age	1.02 (0.98, 1.07)	0.35	1.00 (0.96, 1.04)	0.97	1.02 (0.98, 1.06)	0.29
Baseline EQ5D	3.20 (0.39, 25.98)	0.28	2.35 (0.40, 13.98)	0.35	2.65 (0.32, 21.68)	0.36
Haemorrohoid surgery	3.77 (0.79, 18.00)	0.10	1.32 (0.42, 4.10)	0.63	0.54 (0.15, 2.01)	0.36
Knee arthroscopy	4.02 (1.14, 14.20)	**0.03**	5.22 (1.77, 15.38)	**<0.01**	1.96 (0.65, 5.91)	0.23
Shoulder arthroscopy	54.10 (4.25, 688.25)	**<0.01**	4.93 (1.08, 22.49)	**0.04**	8.40 (1.71, 41.19)	**<0.01**

Data are presented as odds ratio (95% CI). Hosmer and Lemetest shows the goodness of fit of the model (POD 7: p = 0.85, POD 14: p = 0.11 and POD 28: p = 0.87) Nagelkerke R^2^ was calculated (0.29 for POD7, 0.32 for POD 14 and 0.47 for POD28). The false positive rate was 16% for POD7, 20% for POD14 and 26% for POD28. A p-value<0.05 is considered statistically significant.

At POD 14, fear of long-term aspects of surgery and type of surgery were predictors for poor/intermediate QOR. Female gender, having a paid job, postoperative pain on day 4 and type of surgery were found to be predictors for poor/intermediate QOR at day 28.

**Fig 4:**
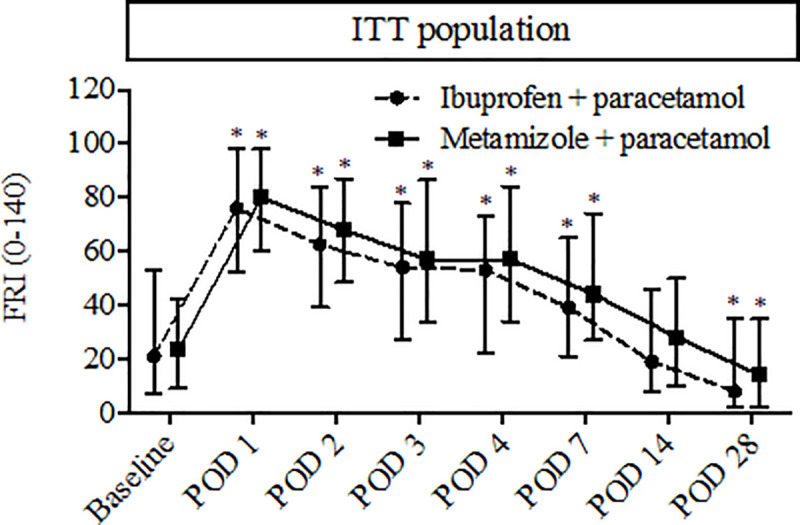
Quality of Recovery (QOR) at baseline and during follow-up. Total functional recovery index (FRI) scores are presented as median (25^th^ - 75^th^ percentile) for the “intention to treat” population. No significant differences were found between pain treatment groups based on the Mann-Whitney U test. QOR at follow-up was also compared to baseline (based on a Wilcoxon signed-rank test and Bonferroni adjustment). Significant differences from baseline are presented with an asterisk (*).

No significant differences between the two different pain treatment groups (metamizole and paracetamol versus ibuprofen and paracetamol) were found during the pain treatment period (Table 5) and at the subsequent follow-up days (Fig 4) for the total FRI scores.

**Table 5:** FRI scores stratified by postoperative pain treatment.

	Ibuprofen + Paracetamol (ITT: n = 98)	Metamizole + Paracetamol (ITT: n = 98)	P-value
**FRI (0–140)**			
**ITT analysis**			
POD 1	76.00 (52.00–98.00)	80.00 (60.00–98.00)	0.383
POD 2	62.50 (39.00–84.00)	68.00 (48.50–87.00)	0.832
POD 3	54.00 (27.00–77.75)	57.00 (33.50–86.50)	0.263

Data are presented as median (25^th^ - 75^th^ percentile). No significant differences between treatment groups were found based on the Mann-Whitney U test. FRI: functional recovery index.

## Discussion

In this secondary analysis of a prospective, randomised controlled trial, we investigated the one-month recovery profile after four types of painful day case surgery. These types of surgery were selected because they are associated with a high incidence of poor QOR at the fourth postoperative day [18]. Furthermore, we assessed the prevalence of poor and good QOR, based on different definitions, and tried to identify predictors of poor QOR. Finally, we assessed the influence of different pain treatment regimens on QOR.

The results of the present study suggest that QOR course varies with type of surgery. For example, baseline scores of FRI and EQ-5D were remarkably worse for patients undergoing arthroscopic shoulder surgery compared with patients undergoing day case haemorrhoid surgery or inguinal hernia repair (Fig 2). Four weeks after arthroscopic shoulder surgery, FRI-, EQ-5D- and GSR- scores were still much worse compared with the other 3 types of day case surgery. These variations in recovery profile are also reflected in the prevalence of good and poor QOR at different time points after surgery. For example, on POD 28, only 24% of patients after arthroscopic shoulder surgery reported good QOR, compared with 71%, 76% and 57% after haemorrhoid surgery, inguinal hernia repair and arthroscopic knee surgery, respectively. However, based on an alternative definition, recovery was incomplete in only 13% to 19% of all patients and improvement was shown in all four surgery groups at POD 28.

Previous studies have already pointed out that the recovery process may take multiple weeks or even months, depending on patient and surgical characteristics and that each type of surgery has its own unique recovery trajectory [3, 4]. Therefore, the International Association for Ambulatory Surgery (IAAS) has recommended that follow-up after day case surgery should at least take place at day 1, day 14 as well as day 30 [3]. We included more assessment points in order to be able to create a better documented recovery profile.

Unfortunately, there are no universally accepted definition and analytic criteria for poor QOR and no universally accepted instrument to measure QOR [27]. As shown in this study, the applied definition of QOR has a major impact on the prevalence of poor and good QOR and as a result, the prevalence of poor QOR varies strongly. Therefore, we suggest the development of a “statement of consensus on the assessment of QOR after day case surgery” by the IAAS. This statement should include information on the recommended time points of assessment, the recommended instruments to assess QOR and the analytic criteria to define poor and good QOR.

The aim of most ambulatory surgical procedures is not to increase life expectancy and certainly not to immediately save a patient’s life but to improve the patient’s QOL. Therefore, a quick recovery after day case surgery is of paramount importance. In this study, improved QOL was found in > 50% of patients four weeks after day case orthopaedic surgery and > 30% of patients four weeks after inguinal hernia repair and after haemorrhoid surgery (Fig 3). These findings can be explained by a lower baseline QOL in our cohort of patients undergoing orthopaedic surgery which can more easily be improved.

Improving QOR implies knowledge of the different variables that influence QOR after day case surgery. Therefore, another primary goal of this study was to identify predictors of poor QOR at POD 7, 14 and 28 after day case surgery. Our data showed that a high postoperative pain level at POD 4 and type of surgery were the most important predictors of poor/intermediate QOR. At POD 7, male gender was also predictive for poor/intermediate QOR. Fear of long-term aspects of surgery was a predictor of poor/intermediate QOR at POD 14. Finally, having a paid job and female gender were independent predictors at POD 28.

The positive correlation between postoperative pain and poor QOR has already been suggested in a previous study. A large prospective cohort study found that patient derived expected pain, which is a good predictor of high postoperative pain levels [28], is also a predictor of poor QOR at POD 4 [18]. This makes sense as postoperative pain plays a pivotal role in the QOR after surgery and is also known to be closely related with not only the physical status but also socio-cultural, cognitive, psychological and functional dimensions [1]. In contrast, we were not able to detect a significant difference in QOR between the two different pain treatment groups (see Table 5). The latter might be explained by the fact that pain levels were similar in both treatment groups [19].

Female gender has already been shown to be a predictor for poor recovery after surgery [29–31]. This may be explained by differences in biology e.g. hormones and genetics [32]. In this study however, we found an association between female gender and long-term (POD 28) poor/intermediate QOR. In contrast, short-term (POD 7) poor/intermediate QOR was associated with male gender. These observations suggest that gender may influence the course of recovery. The association between arthroscopic shoulder surgery and poor/intermediate QOR is already described in literature [18, 33].

E-health, defined as the use of information and communication technologies (ICT) for health, is a promising new strategy to optimize QOR after surgery. Van der Meij et al. [34] reported that a personalized e-health intervention at home after inpatient abdominal surgery can speed up the return to normal activities compared with usual care. Jaensson et al. [35], also proved that a systematic follow-up smartphone-based assessment may increase patients´ QOR after day case surgery. These e-health tools are dependent on data on normal and pathological recovery trajectories after different types of surgery as provided in studies as these. Starting from these data, intervention algorithms may be developed and implemented in an e-health program to improve QOR.

Our study contains some limitations. First, we didn’t evaluate QOR with more surgery-specific instruments. For example, the Constant-Murley score, an instrument to evaluate overall shoulder function, may be more sensitive to assess QOR after day case arthroscopic shoulder surgery than the FRI or EQ-5D [36]. However, we specifically applied those broader tools to make comparison between different types of surgery possible. Second, The response rate of this questionnaire-based decreased with time. Still, telephone follow-up resulted in a 80.5% response rate at four weeks which is better than other questionnaire-based surveys [5]. Third, assessment of postoperative QOR still lacks a standard definition and there is a multitude of QOR measurement tools which make comparison with other studies challenging [27]. Finally, this is a monocentric study. As a result, the generalizability of our results can be questioned.

In conclusion, the present study shows a clear procedure-specific variation in recovery profile in the 4-week period after painful day case surgery. One of the best predictors for short-term (POD 7) and long-term (POD 28) poor/intermediate QOR was a high postoperative pain level at POD 4. Other predictors for poor QOR were having a paid job, fear of long-term aspects of surgery, type of surgery and gender. Different pain treatment regimens didn’t result in differences in QOR (or in postoperative pain level).

## Supporting information

S1 Checklist TREND statement checklist. (DOCX)

S1 Protocol Metamizole versus NSAID at home after ambulatory surgery: A double-blind randomized controlled trial (in Dutch). (DOC)

S2 Protocol Metamizole versus NSAID at home after ambulatory surgery: A double-blind randomized controlled trial (in English). (DOCX)

## List of abbreviations

QOR: Quality of Recovery
NSAID: Non-Steroidal Anti-Inflammatory Drug
FRI: Functional Recovery Index
GSR: Global Surgical Recovery
NRS: Numeric Rating Scale

## References

[1] Wong J, Tong D, De Silva Y, Abrishami A, Chung F. Development of the functional recovery index for ambulatory surgery and anesthesia. *Anesthesiology*. 3 2009;110(3):596–602. 10.1097/ALN.0b013e318197a16d 19212260

[2] Mottram A. "Like a trip to McDonalds": a grounded theory study of patient experiences of day surgery. *International journal of nursing studies*. 2 2011;48(2):165–174. 10.1016/j.ijnurstu.2010.07.007 20678770

[3] Brattwall M, Warren Stomberg M, Rawal N, Segerdahl M, Jakobsson J, Houltz E. Patients' assessment of 4-week recovery after ambulatory surgery. *Acta anaesthesiologica Scandinavica*. 1 2011;55(1):92–98. 10.1111/j.1399-6576.2010.02322.x 21039350

[4] Tran TT, Kaneva P, Mayo NE, Fried GM, Feldman LS. Short-stay surgery: what really happens after discharge? *Surgery*. 7 2014;156(1):20–27. 10.1016/j.surg.2014.03.024 24856316

[5] Halleberg Nyman M, Nilsson U, Dahlberg K, Jaensson M. Association Between Functional Health Literacy and Postoperative Recovery, Health Care Contacts, and Health-Related Quality of Life Among Patients Undergoing Day Surgery: Secondary Analysis of a Randomized Clinical Trial. *JAMA surgery*. 8 1 2018;153(8):738–745. 10.1001/jamasurg.2018.0672 29710226 PMC6584305

[6] Bellani ML. Psychological aspects in day-case surgery. *International journal of surgery*. 2008;6 Suppl 1:S44–46. 10.1016/j.ijsu.2008.12.019 19167936

[7] McIntosh S, Adams J. Anxiety and quality of recovery in day surgery: A questionnaire study using Hospital Anxiety and Depression Scale and Quality of Recovery Score. *International journal of nursing practice*. 2 2011;17(1):85–92. 10.1111/j.1440-172X.2010.01910.x 21251158

[8] Ip HY, Chung F. Escort accompanying discharge after ambulatory surgery: a necessity or a luxury? *Current opinion in anaesthesiology*. 12 2009;22(6):748–754. 10.1097/ACO.0b013e328331d498 19745728

[9] Berg K, Kjellgren K, Unosson M, Arestedt K. Postoperative recovery and its association with health-related quality of life among day surgery patients. *BMC nursing*. 2012;11(1):24 10.1186/1472-6955-11-24 23148514 PMC3534532

[10] Mitchell M. Home recovery following day surgery: a patient perspective. *Journal of clinical nursing*. Feb 2015;24(3–4):415–427. 10.1111/jocn.12615 24811058

[11] White PF, Tang J, Wender RH, et al The effects of oral ibuprofen and celecoxib in preventing pain, improving recovery outcomes and patient satisfaction after ambulatory surgery. *Anesthesia and analgesia*. 2 2011;112(2):323–329. 10.1213/ANE.0b013e3182025a8a 21156974

[12] De Oliveira GS Jr., Fitzgerald P, Streicher LF, Marcus RJ, McCarthy RJ. Systemic lidocaine to improve postoperative quality of recovery after ambulatory laparoscopic surgery. *Anesthesia and analgesia*. 8 2012;115(2):262–267. 10.1213/ANE.0b013e318257a380 22584558

[13] Jakobsson J. Assessing recovery after ambulatory anaesthesia, measures of resumption of activities of daily living. *Current opinion in anaesthesiology*. 12 2011;24(6):601–604. 10.1097/ACO.0b013e32834c3916 21945921

[14] Herrera FJ, Wong J, Chung F. A systematic review of postoperative recovery outcomes measurements after ambulatory surgery. *Anesthesia and analgesia*. 7 2007;105(1):63–69. 10.1213/01.ane.0000265534.73169.95 17578958

[15] Kleinbeck SV. Self-reported at-home postoperative recovery. *Research in nursing & health*. 12 2000;23(6):461–472. 10.1002/1098-240X(200012)23:6&lt;461::AID-NUR5&gt;3.0.CO;2-S 11130605

[16] Royse CF, Newman S, Chung F, et al Development and feasibility of a scale to assess postoperative recovery: the post-operative quality recovery scale. *Anesthesiology*. 10 2010;113(4):892–905. 10.1097/ALN.0b013e3181d960a9 20601860

[17] Mattila K, Lahtela M, Hynynen M. Health-related quality of life following ambulatory surgery procedures: assessment by RAND-36. *BMC anesthesiology*. 2012;12:30 10.1186/1471-2253-12-30 23217178 PMC3556308

[18] Stessel B, Fiddelers AA, Joosten EA, Hoofwijk DM, Gramke HF, Buhre WF. Prevalence and Predictors of Quality of Recovery at Home After Day Surgery. *Medicine*. Sep 2015;94(39):e1553 10.1097/MD.0000000000001553 26426622 PMC4616829

[19] Stessel B, Boon M, Pelckmans C, et al Metamizole vs. ibuprofen at home after day case surgery: A double-blind randomised controlled noninferiority trial. *European journal of anaesthesiology*. 5 2019;36(5):351–359. 10.1097/EJA.0000000000000972 30946703

[20] Stessel B, Boon M, Joosten EA, et al Metamizole versus ibuprofen at home after day surgery: study protocol for a randomised controlled trial. *Trials*. 9 26 2016;17(1):471 10.1186/s13063-016-1586-8 27669689 PMC5037620

[21] Theunissen M, Peters ML, Schouten EG, et al Validation of the surgical fear questionnaire in adult patients waiting for elective surgery. *PloS one*. 2014;9(6):e100225 10.1371/journal.pone.0100225 24960025 PMC4069058

[22] Van Agt HM, Essink-Bot ML, Krabbe PF, Bonsel GJ. Test-retest reliability of health state valuations collected with the EuroQol questionnaire. *Social science & medicine*. 12 1994;39(11):1537–1544. 10.1016/0277-9536(94)90005-1 7817218

[23] Brattwall M, Stomberg MW, Rawal N, Segerdahl M, Houltz E, Jakobsson J. Patient assessed health profile: a six-month quality of life questionnaire survey after day surgery. *Scandinavian journal of public health*. 8 2010;38(6):574–579. 10.1177/1403494810374221 20542959

[24] Suhonen R, Virtanen H, Heikkinen K, et al Health-related quality of life of day-case surgery patients: a pre/posttest survey using the EuroQoL-5D. *Quality of life research*: *an international journal of quality of life aspects of treatment*, *care and rehabilitation*. 2 2008;17(1):169–177. 10.1007/s11136-007-9292-3 18074242

[25] Peters ML, Sommer M, de Rijke JM, et al Somatic and psychologic predictors of long-term unfavorable outcome after surgical intervention. *Annals of surgery*. 3 2007;245(3):487–494. 10.1097/01.sla.0000245495.79781.65 17435557 PMC1877005

[26] Hoofwijk DM, Fiddelers AA, Peters ML, et al Prevalence and Predictive Factors of Chronic Postsurgical Pain and Poor Global Recovery 1 Year After Outpatient Surgery. *The Clinical journal of pain*. 12 2015;31(12):1017–1025. 10.1097/AJP.0000000000000207 25565589

[27] Bowyer A, Jakobsson J, Ljungqvist O, Royse C. A review of the scope and measurement of postoperative quality of recovery. *Anaesthesia*. 11 2014;69(11):1266–1278. 10.1111/anae.12730 24888412

[28] Stessel B, Fiddelers AA, Marcus MA, et al External Validation and Modification of a Predictive Model for Acute Postsurgical Pain at Home After Day Surgery. *The Clinical journal of pain*. 5 2017;33(5):405–413. 10.1097/AJP.0000000000000413 27428546 PMC5638419

[29] Buchanan FF, Myles PS, Cicuttini F. Effect of patient sex on general anaesthesia and recovery. *British journal of anaesthesia*. 6 2011;106(6):832–839. 10.1093/bja/aer094 21558068

[30] Cho CH, Ye HU, Jung JW, Lee YK. Gender Affects Early Postoperative Outcomes of Rotator Cuff Repair. *Clinics in orthopedic surgery*. 6 2015;7(2):234–240. 10.4055/cios.2015.7.2.234 26217471 PMC4515465

[31] Kalkman CJ, Visser K, Moen J, Bonsel GJ, Grobbee DE, Moons KG. Preoperative prediction of severe postoperative pain. *Pain*. Oct 2003;105(3):415–423. 10.1016/S0304-3959(03)00252-5 14527702

[32] Jaensson M, Dahlberg K, Nilsson U. Factors influencing day surgery patients' quality of postoperative recovery and satisfaction with recovery: a narrative review. *Perioperative medicine (London*, *England)*. 2019;8:3 10.1186/s13741-019-0115-1 31139359 PMC6530125

[33] Stessel B, Theunissen M, Marcus MA, et al Prevalence and Predictors of Patient Nonadherence to Pharmacological Acute Pain Therapy at Home After Day Surgery: A Prospective Cohort Study. *Pain practice*: *the official journal of World Institute of Pain*. 2 2018;18(2):194–204. 10.1111/papr.12589 28419729

[34] van der Meij E, Anema JR, Leclercq WKG, et al Personalised perioperative care by e-health after intermediate-grade abdominal surgery: a multicentre, single-blind, randomised, placebo-controlled trial. *Lancet (London*, *England)*. 7 7 2018;392(10141):51–59. 10.1016/S0140-6736(18)31113-9 29937195

[35] Jaensson M, Dahlberg K, Eriksson M, Nilsson U. Evaluation of postoperative recovery in day surgery patients using a mobile phone application: a multicentre randomized trial. *British journal of anaesthesia*. 11 1 2017;119(5):1030–1038. 10.1093/bja/aex331 29077818

[36] Vrotsou K, Avila M, Machon M, et al Constant-Murley Score: systematic review and standardized evaluation in different shoulder pathologies. *Quality of life research*: *an international journal of quality of life aspects of treatment*, *care and rehabilitation*. 9 2018;27(9):2217–2226. 10.1007/s11136-018-1875-7 29748823 PMC6132990

