# Supplementary material for: One-month recovery profile and prevalence and predictors of quality of recovery after painful day case surgery: Secondary analysis of a randomized controlled trial
Source: PLoS One. 2021 Jan 26;16(1):e0245774. doi: 10.1371/journal.pone.0245774 (PMC7837485; doi:10.1371/journal.pone.0245774)
Supplement: S1 Protocol — (DOC) [file pone.0245774.s002.doc]

Metamizole versus NSAID at home after ambulatory surgery: a double-blind randomized controlled trial

Dr. B Stessel

JESSA Ziekenhuis Hasselt

Stadsomvaart 11

3500 Hasselt

Tel.: 0032/479292433

e-mail: bjornstessel@hotmail.com

**INHOUD**

1. INTRODUCTIE EN RATIONALE 4

2. EINDPUNTEN 9

3. STUDY DESIGN 10

4. STUDY POPULATIE [10](#__RefHeading___Toc148844323)

4.1 Populatie [10](#__RefHeading___Toc148844324)

4.2 Inclusie criteria 11

4.3 Exclusie criteria 11

4.4 Stop criteria 11

4.5 Sample size calculatie 12

5. BEHANDELING 13

5.1 Investigational product 13

5.2 Gebruik van co-interventie 13

5.3 Escape medicatie 13

6. ONDERZOEKSMEDICATIE 14

6.1 Naam en beschrijving van onderzoeksmedicatie 14

6.2 Productomschrijving 14

6.3 Risicos van de onderzoeksmedicatie 14

6.4 Beschrijving toediening 16

6.5 Dosering en toediening 16

6.6 Preparatie en labelling van onderzoeksmedicatie 16

6.7 Drug accountability 16

7. METHODEN 18

7.1 Studie parameters/eindpunten 18

7.1.1 Primaire studie parameter/eindpoint 18

7.1.2 Secondaire studie parameters/eindpoints 18

7.1.3 Overige studie parameters 18

7.2 Randomisatie en blindering 18

7.3 Studie procedures 19

7.4 Tussentijds stoppen 21

8. VEILIGHEIDS RAPPORTAGE 21

8.1 Sectie 10 WMO event 21

8.2 Adverse and serious adverse events 22

9. STATISTICAL ANALYSIS 23

9.1 Descriptieve statistics 23

9.2 Analyse 23

9.3 Interim analyse 23

10. ETHISCHE OVERWEGINGEN 24

10.1 Wettelijke voorschriften 24

10.2 Verwerving en instemming 24

10.3 Minderjarigen of wilsonbekamen 25

10.4 Voordelen en risico’s 25

10.5 Verzekering 25

10.6 Vergoedingen 25

11. ADMINISTRATIEVE ASPECTEN EN PUBLICATIE 26

11.1 Hantering en bewaring van data en documenten 26

11.2 Amendementen 26

11.3 Jaarlijkse voortgangsrapportage 26

11.4 Eindrapportage van het onderzoek 27

11.5 Publicatie van de resultaten 27

12. REFERENTIES 28

LIJST VAN AFKORTINGEN

| **NRS** | **Numeric Rating Scale** |
| --- | --- |
| **MIA** | **Metamizole-induced agranulocytosis** |
| **GSR**  **FRI**  **NSAID**  **EQ-5D**  **SFQ** | **Global Surgical Recovery index**  **Functional Recovery Index**  **Non-Steroidal Anti-Inflammatory Drugs**  **Euro Quality Of Life – 5 Dimensions**  **Surgical Fear Questionnaire** |

# INTRODUCTIE EN RATIONALE

Ambulante chirurgie is de laatste tientallen jaren enorm toegenomen en in veel landen behelst het meer dan 50% van alle chirurgie.

Meer complexe chirurgie op oudere patiënten wordt tegenwoordig uitgevoerd door vooruitgang op chirurgisch en anesthesiologisch vlak.

Goede pijnstilling na chirurgie is een belangrijke outcome parameter. Matige tot ernstige pijn kan namelijk leiden tot allerlei fysiologische en psychologische kwalen.

Ondanks de grote aandacht voor pijnstilling na chirurgie blijft postoperatieve pijn een belangrijk probleem. Met name optimale pijnstilling na dagchirurgie is erg complex omdat de patiënten in deze setting reeds na een aantal uren worden onttrokken aan de zorg van medisch personeel. Hierdoor is verlengde postoperatieve pijnstilling door middel van intraveneuze pijnpompen, epidurale of perifere zenuwcatheters geen realistische mogelijkheid. Ook subcutane of intramusculair geplaatste sterke opioiden zijn geen realistische optie gezien het ontbreken van geschoold medisch personeel om deze opioiden toe te dienen en gezien het mogelijke risico op ademhalingsdepressie in een niet-veilige omgeving. Gezien deze overwegingen is de gouden standaard van pijnstilling thuis na ambulante chirurgie op dit ogenblik een multimodaal pijnregime bestaande uit een combinatie van een Non-Steroidal Anti-Inflammatory Drug (NSAID) met paracetamol, al dan niet aangevuld met een zwak opioid zoals tramadol. (Tevens wordt de chirurg aangespoord om de wonde te infiltreren met een lokaal anesthesteticum. Specifiek voor schouderchirurgie kan ook geopteerd worden voor een plexus brachialisblok. Echter deze infiltraties met lokaal anestheticum hebben slechts een werkingsduur van 8 tot 24u.

Ondanks deze multimodale pijntherapie blijft matig tot ernstige pijn na dagchirurgie een groot klinisch probleem na bepaalde types van ambulante chirurgie.

Met name anale chirurgie, schouderchirurgie, scopie knie, tandchirurgie, liesbreukherstel en dupuytrenchirurgie blijken zelfs op dag 4 na ambulante chirurgie nog gepaard te gaan met gemiddeld matige tot ernstige pijn.

De afgelopen jaren is herstel in een meer brede betekenis een belangrijke outcome parameter geworden van ambulante chirurgie. Dit herstel behelst meerdere dimensies en om dit te meten zijn er een aantal gevalideerde instrumenten beschikbaar. Hiermee kan herstel vroeg (nog in het ziekenhuis), intermediair (dag 3 – 7) of laat (na 1 week) gemeten worden. Uiteraard is pijn op zich een belangrijke dimensie van herstel en kan pijn een belangrijke invloed hebben op andere dimensies van herstel.

Dipyrone of Metamizole is een niet-opioid met sterke analgetische, antipyretische en spasmolytische effecten ^1^. Het werd voor het eerst op de markt gebracht in Duitsland in 1922 ^2^. Het analgetische en antipyretische werkingsmechanisme is tot op heden nog niet volledig uitgeklaard. Dipyrone is een pro-drug. Het dankt zijn analgetische en antipyretische activiteit aan een snelle conversie naar zijn actieve metabolieten ^3^. De afgelopen jaren zijn er meerdere werkingsmechanismes voorgesteld: COX-1/COX-2 inhibitie door dipyrone en zijn metabolieten enerzijds en de vrijzetting van endogene opioiden anderzijds ^4,5^. Recent is er ook toenemende evidentie dat het endocannabinoid/endovanilloid systeem een belangrijke rol speelt in het analgetisch effect van dipyrone ^2,3,6,7^. Dit endocannabionoid systeem is de laatste jaren steeds meer in de aandacht gezien het een opportuniteit biedt tot ontwikkeling van multitarget analgetica ^8^. Co-administratie van tramadol en dipyrone zou een belangrijk synergistisch effect hebben op hun individuele antinociceptieve werking ^9,10^.

Een ander niet onbelangrijk voordeel van dipyrone is het gunstige gastro-intestinale ^11,12^ en mogelijk ook cardiovasculaire en cerebrovasculaire profiel ten opzichte van NSAID´s ^13-17^.

In contrast hiermee wordt het gebruik van dipyrone ter discussie gesteld en is het zelfs verboden in meerdere landen inzake zijn associatie met agranulocytose. Deze restricties zijn gebaseerd op 2 studies welke wezen op een zeer hoge incidentie van Metamizole-geïnduceerde agranulocytose (MIA) ^18,19^.

Echter, meer recente literatuur staaft het idee dat de incidentie van MIA zeer gering is, ergens in de grootte-orde van 0,5 tot 1 casus per miljoen per jaar ^1,20-23^. Bovendien heeft Andrade et al. aangetoond dat de mortaliteit ten gevolge van agranulocytose, aplastische anemie, anaphylaxie en gastro-intestinale complicaties in het voordeel is van dipyrone vergeleken met diclophenac (25 per 100 million vs 592 per 100 million) ^24^.

In de literatuur is de analgetische doeltreffendheid van intraveneus of intramusculair toegediende dipyrone voor pijnstilling na electieve chirurgie reeds uitvoerig beschreven ^25-31^.

Rawal heeft ook reeds de analgetische werkzaamheid van tramadol, paracetamol en dipyrone vergeleken voor postoperatieve pijnstilling thuis na ambulante handchirurgie ^32^. Echter, tegenwoordig wordt een mulitmodaal analgetisch regime, bestaande uit een combinatie van paracetamol, NSAID´s, zwakke opioiden en lokale of locoregionale anesthetica bepleit voor pijnstilling na ambulante chirurgie ^33-35^. Bovendien heeft Ong in een systematic review reeds aangetoond dat een combinatie van paracetamol en een NSAID een superieur analgetisch effect heeft vergeleken met het analgetisch effect van elk medicament apart ^36^.

In het JESSA-ziekenhuis bestaat het standaard multimodale pijnregime voor pijnstilling thuis na ambulante chirurgie uit een combinatie van ibuprofen, een NSAID met een gunstige analgetisch en bijwerkingsprofiel, en paracetamol ^37^. Tramadol wordt hierboveop vaak gebruikt als rescue-analgeticum.

Het primaire doel van deze studie is tweeledig:

1. Het bestuderen en vergelijken van de analgetische kracht van een combinatie van paracetamol en metamizole met het standaard pijnprotocol (paracetamol/ibuprofen) in een groep van patiënten die pijnlijke ambulante chirurgie ondergaan. Onze hypothese is dat de combinatie van paracetamol met metamizole minstens een even grote pijnreductie geeft vergeleken met het huidige standaard pijn protocol.
2. Het bestuderen en vergelijken van het herstelprofiel van de twee behandelgroepen en van verschillende types van pijnlijke ambulante chirurgie.

Secundaire onderzoeksdoelen zijn het nagaan van eventuele nadelige effecten van studiemedicatie, het gebruik van rescue-medicatie (dipidolor iv in recovery en tramadol oraal thuis), het bestuderen van de patiënttevredenheid, de compliantie met studiemedicatie en predictoren van postoperatieve pijn en herstel.

**Hypotheses**

1. De combinatie metamizole en paracetamol geeft een even grote of grotere reductie van postoperatieve pijn ten opzichte van de combinatie ibuprofen en paracetamol.
2. Ieder type van ambulante chirurgie heeft een uniek herstelprofiel, significant verschillend van het herstelprofiel van de andere types van ambulante chirurgie.
3. De combinatie metamizole en paracetamol geeft een even goed of beter herstel na ambulante chirurgie ten opzichte van de combinatie ibuprofen en paracetamol.
4. Er worden weinig of geen neveneffecten gezien bij het gebruik van perorale metamizole in kader van postoperatieve pijnstilling na ambulante chirurgie
5. De patiënttevredenheid is hoog in beide behandelgroepen

# EINDPUNTEN

**Primaire eindpunten**

- Postoperatieve pijnintensiteit gemeten door NRS.

Vraagstelling: Is er een verschil in postoperatieve pijnintensiteit in de verschillende behandelgroepen? Een verschil van meer dan 1 punt op de NRS wordt als klinisch relevant beschouwd. Dit verschil wordt bewust zo klein gehouden omdat het een non-inferiority trial betreft.

- Postoperatief herstel gemeten door de GSR ^38,39^, de verschil-score van de EQ-5D ^40^ en de FRI ^41,42^.

Vraagstelling: Is er een verschil in herstel in de verschillende behandelgroepen en tussen de verschillende types ambulante chirurgie, gemeten met de 3 verschillende meetinstrumenten?

**Secundaire eindpunten**

- Neveneffecten van de gebruikte analgetica: pyrosis, tekens van agranulocytosis of thrombocytopenie
- Patiënttevredenheid
- Gebruik van rescue-medicatie (dipidolor in recovery en tramadol thuis)
- Compliantie met studiemedicatie thuis
- Predictoren van postoperatieve pijn en herstel

# STUDY DESIGN

Het betreft een monocentrisch, dubbelblind, prospectief, gerandomiseerd onderzoek, waarbij 2 behandelgroepen worden vergeleken. Het betreft een non-inferiority trial.

Groep 1: postoperatieve. dagen 1, 2, 3, 4: paracetamol 1000mg 4x/dag, ibuprofen 600mg 3x/ dag (standaard JESSA ziekenhuis)

Groep 2: postoperatieve. dagen 1, 2, 3, 4: paracetamol 1000mg 4x/dag, metamizole 1000mg 3x/dag

**Steekproeftrekking:**

Randomisatie vindt plaats middels een van tevoren door de computer gegenereerde lijst. Per type chirurgie wordt een aparte randomisatielijst aangemaakt, gezien 4 types chirurgie worden geïncludeerd worden 4 lijsten aangemaakt.

**Tijdschema:**

Het onderzoek duurt ongeveer 6 maanden, afhankelijk van het aanbod van deze patiënten kan deze termijn langer duren (maximaal 8 maanden).

# STUDIE POPULATIE

## Populatie

De studie betreft patiënten die op het dagcentrum ambulant geopereerd worden voor anale pathologie (enkel haemorrhoïden), arthroscopie knie, arthroscopie schouder of liesbreukherstel.

Er is gekozen voor deze pathologie:

- om enerzijds de 2 studiegroepen zo homogeen mogelijk te maken
- omdat eerder onderzoek in het MUMC heeft uitgewezen dat patiënten die 1 van deze ingrepen ondergaan, op dag 4 na de ingreep nog gemiddeld 3 of zelfs 4 scoren op de NRS-pijnscore (dus gemiddeld nog moderate pijn hebben).

## Inclusie criteria

- Patiënten in de leeftijdscategorie ≥18 jaar en ≤70jaar
- ASA classificatie 1,2 of 3
- 1 van de volgende ingrepen: Anale chirurgie (Haemorrhoïden), scopie knie en schouder en liesbreukherstel
- Gewicht > 50kg en < 100kg

## Exclusie criteria

- Leeftijd <18 jaar en >70 jaar
- Onmogelijke toepassing van de NRS voor de meting van de pijnintensiteit. Vb: visuele dysfunctie, dementie
- Geen beheersing van de Nederlandse taal
- Preoperatieve therapie met opioïden
- Fibromyalgie, CRPS, chronische pijn
- Overgevoeligheid voor metamizole, alcohol, paracetamol of ibuprofen (en andere NSAIDs)
- Porphyrie
- Congenitaal tekort aan glucose-6-fosfaat dehydrogenase
- Zwangerschap en borstvoeding
- Ernstige nier- en leverfunctiestoornissen
- Astma
- Rhinosinusitis of neuspoliepen
- Ernstige COPD, emfyseem
- Chronische obstipatie
- Gebruik van anti-rheumatische middelen
- Hematologische ziekte
- Hypotensie
- Ulcus pepticum, maagdarmbloedingen: actief of in de anamnese
- Gastro-intestinale bloeding of perforatie als gevolg van gebruik van cyclooxygenase-inhibitoren in de anamnese
- Ernstig hartfalen
- Opioidabusus in de anamnese
- Chronisch alcoholgebruik
- Gebruik van centraal depressieve stoffen zoals benzodiazepines
- Koorts of andere tekens van acute infectie
- Specifiek voor arthroscopie schouder: niet plaatsen van een interscalenusblok

## Stopcriteria

- - wens van de patiënt de studie af te breken
  - chirurgische complicatie die revisieoperatie of opname noodzakelijk maakt

## Sample size calculatie

De primaire uitkomstmaten zijn de postoperatieve pijnintensiteit, gemeten met de NRS (schaal 0 tot 10) en het postoperatief herstelpatroon. Gezien het herstelpatroon met name beïnvloed zal worden door de postoperatieve pijnintensiteit, zal de sample size calculatie gebeuren op basis van de verwachte pijnintensiteit.

Op grond van eerdere studies ^43,44^ is de standaarddeviatie van de NRS van dag 1 tot en met 4 postoperatief na ambulante chirurgie op maximaal 2,5 geschat (en minimaal 1,7 = dag 4). Om, uitgaande van een SD = 2,5, met een power van 80% en een significantieniveau van 0,05 (1-zijdig) een verschil van meer dan één punt op de NRS tussen twee de behandelgroepen uit te sluiten, zijn per groep 78 patiënten nodig. Rekening houdend met een drop-out rate tot 25%, zullen we per behandelgroep 100 patiënten includeren.

# BEHANDELING

## Investigational product

Postoperatieve analgesie met metamizole in combinatie met paracetamol (zie 7.3.)

## Gebruik van co-interventie

Niet van toepassing

## Escape medicatie

Escapemedicatie groepen 1 en 2: Tramadol odis 50mg

# ONDERZOEKSMEDICATIE

## Naam en beschrijving van onderzoeksmedicatie

Metamizole:

metamizole is een analgeticum waarvan het werkingsprofiel uniek is (zie rationale).

Paracetamol:

Paracetamol is een niet-opioïd analgeticum.

Ibuprofen:

Ibuprofen hoort bij de groep van de niet-steroïdale anti-inflammatoire middelen (NSAIDs).

## Productomschrijving

Zie bijlage 1 (PDF IB1Metamizole)

Zie bijlage 2 (PDF IB1Paracetamol1000)

Zie bijlage 3 (PDF IB1Ibuprofen)

## Risico´s van de onderzoekmedicatie

Paracetamol:

Paracetamol is bestanddeel van het standaard pijnprotocol voor postoperatieve analgesie in het JESSA-ziekenhuis. Het risico voor de patiënt wordt door deelname aan dit onderzoek niet verhoogd. In therapeutische doseringen zoals gebruikt in dit onderzoek treden weinig bijwerkingen op. Overgevoeligheidsreacties meestal in de vorm van exantheem en urticaria. Koorts, thrombocytopenie en hemolytische anemie kunnen optreden, maar zijn zeldzaam. Ernstige bijwerkingen en complicaties treden met name bij toxische dosering en langdurig gebruik op. In het kader van dit onderzoek wordt paracetamol kortdurend gebruikt (96 uren). Voor een uitgebreide beschrijving van de bijwerkingen wordt naar verwezen naar bijlage 2. In de exclusiecriteria wordt rekening gehouden met de bijwerkingen en contra-indicaties.

Ibuprofen:

Ibuprofen wordt als standardanalgeticum voor postoperatieve analgesie op het dagcentrum van het JESSA-ziekenhuis gebruikt, rekening houdend met de contra-indicaties. Het risico voor de patiënt wordt door deelname aan dit onderzoek niet verhoogd. In het kader van het onderzoek gaat het om kortdurend gebruik (96 uren). Overgevoeligheidsreacties zijn mogelijk, met name allergische huidreacties (urticaria, pruritus). Gastro-intestinale bijwerkingen zijn relatief frequent, zuurbranden, abdominale pijn, misselijkheid, obstipatie. Ernstige gastro-intestinale bijwerkingen zoals bloedverlies uit het maag-darmkanaal, ulcus pepticum, perforatie van het maag-darmkanaal en andere zijn minder frequent en correleren met de behandelingsduur. Verder heeft ibuprofen een inhiberend effect op de thrombocytenaggregatie met verlengde bloedingstijd. Nierfunctiestoornissen, longoedeem en hartfalen zijn ook mogelijk. Voor een uitgebreide beschrijving van de bijwerkingen wordt naar verwezen naar bijlage 3. In de exclusiecriteria wordt rekening gehouden met de bijwerkingen en contra-indicaties.

Metamizole

Metamizole hoort niet bij de standaardanalgetica voor postoperatieve pijnbehandeling in het JESSA-ziekenhuis. In het kader van het onderzoek gaat het om kortdurend gebruik (96 uren). Overgevoeligheidsreacties zijn mogelijk, met name allergische huid- en slijmvliesreacties (erytheem, urticaria, pruritus, aften, blaren) alsook bronchospasme en zeer zeldzaam anaphylactoïde shock. Verder is last ter hoogte van maag-darmkanaal mogelijk. Tevens zal het gebruik van metamizole de urine rood kleuren aangezien metamizole wordt omgezet in een product met een rode kleur. Dit is niet schadelijk. In geïsoleerde gevallen en in gevallen van overdosering zijn reversibele nierfunctiestoornissen mogelijk. Zeer zeldzaam kan metamizole leiden tot agranulocytosis welke zich manifesteert door optreden van sepsis (koorts, rillingen en andere tekens van infectie) en heftige keelpijn (angina) en aften in de mond. Tevens zeer zeldzaam kan thrombocytopenie voorkomen welke zich manifesteert door verhoogde bloedingsneiging of puntbloedingen ter hoogte van huid.

Occasioneel kan er zich een geïsoleerde bloeddrukdaling (hypotensie) voordoen, niet gerelateerd aan een allergische reactie, met name bij intraveneuze toediening.

## Beschrijving toediening

Metamizole wordt in deze studie in tabletvorm oraal toegediend.

Paracetamol wordt in deze studie in tabletvorm oraal toegediend.

Ibuprofen wordt in deze studie in tabletvorm oraal toegediend.

## Dosering en toediening

Zie 7.3 voor doseringen

De gekozen dosering van metamizole komt overeen met bestaande literatuur over metamizole voor postoperatieve pijnstilling.

De gekozen dosering van paracetamol komt overeen met de sinds jaren gebruikelijke dosering op het dagcentrum van het JESSA ziekenhuis en is eveneens congruent met de medische literatuur.

De gekozen dosering van ibuprofen komt overeen met de sinds jaren gebruikelijke dosering op het dagcentrum van het JESSA ziekenhuis en is eveneens congruent met de medische literatuur.

## Preparatie en labelling van onderzoeksmedicatie

Normale standaardverpakking voor Paracetamol

Geblindeerde verpakking (door apotheek) voor Ibuprofen en Metamizole

## Drug accountability

De studiemedicatie zal door de apotheek in blisterverpakking afgeleverd worden. Elk doosje bevat de studiemedicatie voor een patiënt voor de hele studieperiode (96 uren postoperatief). Bij de twee behandelingsgroepen zullen dus twee verschillende soorten doosjes gemaakt worden. De randomisatielijst bepaalt welke patiënt welke studiemedicatie krijgt.

# METHODEN

## Studie parameters/eindpunten

### Primaire studie parameter/eindpunten

Postoperatieve pijnintensiteit gemeten door NRS.

Postoperatief herstel, gemeten door GSR, EQ-5D en FRI.

### Secundaire studie parameters/eindpunten

Neveneffecten van de gebruikte analgetica: pyrosis, tekens van agranulocytosis of thrombocytopenie, enz

Patiënttevredenheid

Gebruik van rescue-medicatie (dipidolor in recovery en tramadol thuis)

Compliantie met studiemedicatie thuis

Predictoren van postoperatieve pijn en herstel

### Overige studie parameters

Prevalentie van NRS > 30mm (cut-off waarde die in het JESSA ziekenhuis gehandhaafd wordt voor onacceptabele pijn)

## Randomisatie en blindering

Randomisatie vindt plaats middels een van tevoren door de computer gegenereerde lijst. Per type chirurgie wordt een aparte randomisatielijst aangemaakt, gezien 4 types chirurgie worden geïncludeerd worden 4 lijsten aangemaakt van 50 patiënten (telkens 2x25 patiënten).

Het gaat om een dubbel geblindeerde studie.

De studiemedicatie (Metamizole en Ibuprofen) zal door de apotheek geblindeerd in kartonnen doosjes afgeleverd worden (medicatie A en medicatie B). Elk doosje bevat de studiemedicatie voor een patiënt voor de hele studieperiode (96 uren postoperatief). De randomisatielijst bepaalt welke patiënt welke studiemedicatie krijgt. Enkel de hoofdonderzoeker (Dr. Stessel) heeft toegang tot deze lijst. Onderzoekers die de outcome parameters verzamelen zijn net zoals de patiënten geblindeerd.

## Studie procedures

**Pre-operatieve fase:**

De informatiebrief en het informed consent formulier wordt reeds tijdens de preoperatieve consultatie door de chirurg meegegeven aan de patiënt.

Bij aankomst op het dagkliniek wordt elke patiënt die het informed consent formulier heeft ondertekend, geïncludeerd in de studie, nadat is nagegaan of er geen exclusiecriteria zijn. Op basis van de randomisatielijst wordt de patiënt ingedeeld in 1 van de 2 studiegroepen. De NRS-score en het gebruik van het pijndagboek worden in detail uitgelegd door een lid van het onderzoeksteam, waarna de patiënt de baselinegegevens invult op het case report form (CRF). Dit bestaat uit de demografische gegevens (leeftijd, gewicht, lengte, geschiedenis van PONV of wagenziekte), de pre-operatieve NRS-score, de EQ-5D en de FRI. Tevens wordt de surgical fear questionnaire afgenomen, nagegaan hoeveel pijn de patiënt verwacht te hebben postoperatief en welke de werkstatus en opleidingsniveau is van de patiënt. De gedetailleerde medische voorgeschiedenis wordt in de pre-operatieve screening nagegaan (gerelateerde en niet gerelateerde chirurgie wordt actief bevraagd).

Nadat de baseline-gegevens zijn geregistreerd, wordt preoperatief paracetamol 1000mg per os aan alle patiënten toegediend. Afhankelijk van de studiegroep, wordt tevens ibuprofen 600mg per os dan wel metamizole 1000mg per os toegediend.

Bij elke patiënt die een arthroscopie schouder ondergaat en meedoet aan de studie, wordt een interscalenusblok geplaatst. Niet plaatsen van een interscalenusblok is dus een exclusiecriterium.

**Per-operatieve fase:**

Elke patiënt ondergaat de narcose op gestandaardiseerde wijze. Deze bestaat uit een inductie met propofol 2mg/kg iv, rapifen 10mcg/kg iv en sufentanil 0,15mcg/kg iv. Na plaatsen van een larynxmasker (of intubatie in het geval van scopie schouder -> uiteraard na toediening rocuronium 20-40mg)) wordt de narcose onderhouden met sevoflurane en een lucht/zuurstof mengsel. Tevens krijgt elke patiënt PONV-profylaxe onder vorm van odansetron 4mg iv. De duur van de operatie zal worden bijgehouden.

**Postoperatieve fase:**

Op de recovery worden alle patiënten, indien de NRS > 30mm gemeten wordt, intraveneus opgeladen met piritramide tot NRS ≤ 30mm.

Doseringsregimes postoperatieve analgesie:

Groep 1: postop. dagen 1, 2, 3, 4: paracetamol 1000mg 4dd, ibuprofen 600mg 3dd (standaard JESSA ziekenhuis)

Groep 2: postop. dagen 1, 2, 3, 4: paracetamol 1000mg 4dd, metamizole 1000mg 3dd

Escapemedicatie groepen 1 en 2: tramadol 3x100mg/d

Pijnintensiteit:

NRS wordt op de recovery en voor ontslag naar huis gemeten.

De verbruikte hoeveelheid dipidolor wordt geregistreerd.

De patiënten wordt een dagboek meegegeven, waarin 1 keer per dag de NRS in rust en de NRS bij beweging ingevuld wordt om 12u. (dag 1 t.e.m. 4, dag 7, dag 14 en dag 28).

Kwaliteit van herstel en patiënttevredenheid:

Op dag 1 t.e.m 4, dag 7, dag 14 en dag 28 wordt de de GSR, de post-op EQ-5D en de FRI gescoord.

Tevens wordt op dag 7 de patiënttevredenheid gescoord.

Om de loss to follow-up zo laag mogelijk te houden worden de patiënten telefonisch herinnerd aan hun deelname tot studie op de volgende dagen: dag 1 t.e.m. 4, dag 7, dag 14 en dag 28 na de ingreep. De gegevens worden dan telefonisch opgevraagd.

Veiligheid:

Patiënten worden verwittigd dat er een zeer kleine kans bestaat dat zich een agranulocytosis voordoet welke zich manifesteert door optreden van sepsis (koorts, rillingen en andere tekens van infectie) en heftige keelpijn (angina) en aften in de mond. Tevens worden zij verwittigd dat er een zeer kleine kans bestaat op thrombocytopenie welke zich manifesteert door verhoogde bloedingsneiging of puntbloedingen ter hoogte van huid. De patiënten worden gevraagd zich via spoed aan te melden en actief te vragen naar Dr. Stessel en/of Dr. Theunissen. De patiënten worden tevens op de volgende tijdstippen gecontacteerd waarbij actief naar eventuele symptomen van beenmergsuppressie wordt gevraagd: d1 t.e.m 4, d7, d14 en d28 postoperatief.

## Tussentijds stoppen

Patiënten kunnen uit de studie stappen op elk tijdstip en voor elke reden zonder enige negatieve consequentie. De onderzoeker kan beslissen een patiënt uit de studie te excluderen vanwege urgente medische redenen. De gegevens van patiënten die na randomisatie afvallen worden in de analyse meegenomen tot aan het tijdstip van uitstappen uit de studie.

# VEILIGHEIDS RAPPORTAGE

## Sectie 10 WMO event

In overeenstemming met sectie 10, subsectie 1, van de WMO, “the investigator will inform the subjects and the reviewing accredited METC if anything occurs, on the basis of which it appears that the disadvantages of participation may be significantly greater than was foreseen in the research proposal. The study will be suspended pending further review by the accredited METC, except insofar as suspension would jeopardise the subjects’ health. The investigator will take care that all subjects are kept informed.

## Adverse and serious adverse events

Adverse events worden gedefinieerd als elke onwenselijke ervaring optredend bij een proefpersoon gedurende een klinische trial, al dan niet gerelateerd aan de onderzoeksmedicatie. Alle adverse events spontaan gerapporteerd door proefpersoon of waargenomen door de onderzoeker of het onderzoeksteam, zullen gerapporteerd worden.

Een Serious Adverse Event (SAE) is enig ongewenst medisch voorval dat

- resulteert in dood
- levensbedreigend (gedurende enig tijdstip) is
- de ziekenhuisopname of verlenging van opnameduur verlengt
- resulteert in aanhoudende of significante handicap of gebrek
- een aangeboren anomalie of geboorteafwijking is
- waarschijnlijk invloed heeft op de veiligheid van de proefpersonen, zoals een onverwachte outcome van een bijwerking, belangrijke veiligheidsuitkomst gevonden van een recent afgeronde dierenstudie, etc.

Alle SAE’s zullen gerapporteerd worden aan de Ethische toetsingscommissie (ETC), welke het protocol heeft goedgekeurd, volgens de richtlijnen van deze ETC.

# STATISTICAL ANALYSIS

## Descriptieve statistics

Continue variabelen worden beschreven met gemiddelden en standaard deviaties, en categorische variabelen met proporties en 95% betrouwbaarheidsinterval.

## Analyse

Voor de vergelijking ibuprofen versus metamizole zullen de NRS scores en de herstelscores tussen de studiegroepen univariaat door middel van t-toets en multivariaat door middel van lineaire regressie worden vergeleken.

## Interim analyse

Niet van toepassing

# ETHISCHE OVERWEGINGEN

## Wettelijke voorschriften

De studie zal uitgevoerd worden geheel volgens:

**“WORLD MEDICAL ASSOCIATION DECLARATION OF HELSINKI**

**Ethical Principles for Medical Research Involving Human Subjects**

Adopted by the 18th WMA General Assembly, Helsinki, Finland, June 1964, and amended by

the

29th WMA General Assembly, Tokyo, Japan, October 1975

35th WMA General Assembly, Venice, Italy, October 1983

41st WMA General Assembly, Hong Kong, September 1989

48th WMA General Assembly, Somerset West, Republic of South Africa, October 1996

and the 52nd WMA General Assembly, Edinburgh, Scotland, October 2000

Note of Clarification on Paragraph 29 added by the WMA General Assembly, Washington 2002

Note of Clarification on Paragraph 30 added by the WMA General Assembly, Tokyo 2004”

## Verwerving en instemming

Minstens 24 uren preoperatief zullen de patiënten mondeling en schriftelijk over de studie geïnformeerd worden door een lid van het onderzoeksteam of de researchverpleegkundige van de afdeling anesthesiologie. Deze informatie gaat principieel bij de preoperatieve chirurgische raadpleging verstrekt worden. De bedenktijd loopt tot maximaal aan de dag van de operatie, in elk geval dus meer dan 24 uren. Door een informed consent te tekenen verlenen de patiënten hun toestemming voor de inclusie in de studie. Daadwerkelijk tekenen van het informed consent door de patiënt is nog op de dag van de operatie mogelijk vóór de toediening van premedicatie. De deelname aan de studie is absoluut vrijwillig en de patiënt heeft op elk moment tijdens de studie de mogelijkheid om zijn beslissing te veranderen. De kwaliteit van zorg wordt hierdoor niet beïnvloed. Patientengegevens worden vertrouwelijk behandeld en zullen alleen (gecodeerd) beschikbaar zijn voor het onderzoeksteam.

## Minderjarigen of wilsonbekwamen

Niet van toepassing

## Voordelen en risico’s

De prevalentie van postoperatieve pijn na ambulante heelkunde is onveranderd hoog. De toevoeging van metamizole zou tot een verbetering van de postoperatieve analgesie kunnen leiden. Bijwerkingen zijn in de regel mild. De meest ernstige bijwerking is agranulocytosis doch deze bijwerking is zeer zeldzaam ^1,20,45^.

## Verzekering

## Vergoedingen

Geen

# PUBLICATIE

## Hantering en bewaring van data en documenten

Gegevens en data van de patiënten worden vertrouwelijk behandeld. De hardcopies van de CRF’s worden in een gesloten kast bewaard en zijn alleen toegankelijk voor leden van het onderzoeksteam. Data worden geanonimiseerd elektronisch ingevoerd voor verdere statistische analyse. De hardcopies van de CRF’s en de analyse zullen minimaal 15 jaren bewaard worden.

## Amendementen

Substantieel amendement wordt gedefinieerd als een amendement van de voorwaarden van de ETC aanvraag, het onderzoeksprotocol zelf of andere ondersteunende documentatie, die waarschijnlijk een significant invloed heeft op :

- - de veiligheid of fysieke of mentale integriteit van de deelnemende patiënten
  - de wetenschappelijke waarde van het onderzoek
  - de uitvoering of management van het onderzoek, of
  - de kwaliteit of veiligheid van enige interventie gebruikt in het onderzoek

Alle substantiële amendementen worden aan de ETC en het FAGG voorgelegd.

Niet-substantiële amendementen worden niet aan de ETC en CCMO gemeld. Deze worden alleen schriftelijk gedocumenteerd en bewaard.

## Jaarlijkse voortgangsrapportage

De hoofdonderzoeker gaat jaarlijks een kort rapport over de vooruitgang van het onderzoek aan de ETC afleveren. Dit report gaat informatie bevatten over de datum van de inclusie van de eerste patiënt, het aantal geïncludeerde patiënten, het aantal patiënten, die het onderzoek afgerond hebben, “serious adverse events”, andere problemen en amendementen.

## Eindrapportage van het onderzoek

Na einde van het onderzoek gaat de hoofdonderzoeker de ETC en het FAGG binnen de 90 dagen op de hoogte stellen. Einde van het onderzoek wordt gedefinieerd als het laatste telefonisch consult (28 dagen na de chirurgische ingreep) van de laatste patiënt.

Bij voortijdige beëindiging van het onderzoek gaat de hoofdonderzoeker de ETC en het FAGG binnen de 15 dagen verwittigen. De redenen van het voortijdig beëindigen van de studie zal ook meegedeeld worden.

Binnen een jaar na beëindiging van de studie wordt een eindrapportage van het onderzoek aan de ETC en het FAGG afgeleverd. Hierin worden ook publicaties/abstracts van de studie meegedeeld. In het geval dat de eindrapportage na verloop van een jaar na beëindiging van het onderzoek nog niet beschikbaar is, wordt een andere termijn afgesproken. De redenen voor het uitstel van de eindrapportage moeten aangegeven worden.

## Publicatie van de resultaten

De resultaten van dit onderzoek zullen bekend gemaakt worden (onafhankelijk van de uitkomst). De voorkeur gaat hierbij naar een “peer-reviewed” wetenschappelijke tijdschrift. Instituut en /of onderzoeker hebben het recht tot publicatie van de resultaten van deze studie. Copyright op publicaties gemaakt door de Onderzoeker, blijven eigendom van de Onderzoeker.

**12. REFERENTIES**

1. Huber M, Andersohn F, Sarganas G, et al. Metamizole-induced agranulocytosis revisited: results from the prospective Berlin Case-Control Surveillance Study. *European journal of clinical pharmacology.* Feb 2015;71(2):219-227.

2. Rogosch T, Sinning C, Podlewski A, et al. Novel bioactive metabolites of dipyrone (metamizol). *Bioorganic & medicinal chemistry.* Jan 1 2012;20(1):101-107.

3. Maione S, Radanova L, De Gregorio D, et al. Effects of metabolites of the analgesic agent dipyrone (metamizol) on rostral ventromedial medulla cell activity in mice. *European journal of pharmacology.* Feb 5 2015;748:115-122.

4. Pierre SC, Schmidt R, Brenneis C, Michaelis M, Geisslinger G, Scholich K. Inhibition of cyclooxygenases by dipyrone. *British journal of pharmacology.* Jun 2007;151(4):494-503.

5. Vanegas H, Tortorici V. Opioidergic effects of nonopioid analgesics on the central nervous system. *Cellular and molecular neurobiology.* Dec 2002;22(5-6):655-661.

6. dos Santos GG, Dias EV, Teixeira JM, et al. The analgesic effect of dipyrone in peripheral tissue involves two different mechanisms: neuronal K(ATP) channel opening and CB(1) receptor activation. *European journal of pharmacology.* Oct 15 2014;741:124-131.

7. Crunfli F, Vilela FC, Giusti-Paiva A. Cannabinoid CB1 receptors mediate the effects of dipyrone. *Clinical and experimental pharmacology & physiology.* Mar 2015;42(3):246-255.

8. Maione S, Costa B, Di Marzo V. Endocannabinoids: a unique opportunity to develop multitarget analgesics. *Pain.* Dec 2013;154 Suppl 1:S87-93.

9. Moreno-Rocha LA, Dominguez-Ramirez AM, Cortes-Arroyo AR, Bravo G, Lopez-Munoz FJ. Antinociceptive effects of tramadol in co-administration with metamizol after single and repeated administrations in rats. *Pharmacology, biochemistry, and behavior.* Nov 2012;103(1):1-5.

10. Montes A, Warner W, Puig MM. Use of intravenous patient-controlled analgesia for the documentation of synergy between tramadol and metamizol. *British journal of anaesthesia.* Aug 2000;85(2):217-223.

11. Batu OS, Erol K. The effects of some nonsteroidal anti-inflammatory drugs on experimental induced gastric ulcers in rats. *Inflammopharmacology.* Dec 2007;15(6):260-265.

12. Yildirim E, Sagiroglu O, Kilic FS, Erol K. Effects of nabumetone and dipyrone on experimentally induced gastric ulcers in rats. *Inflammation.* Apr 2013;36(2):476-481.

13. Castellsague J, Riera-Guardia N, Calingaert B, et al. Individual NSAIDs and upper gastrointestinal complications: a systematic review and meta-analysis of observational studies (the SOS project). *Drug safety.* Dec 1 2012;35(12):1127-1146.

14. Chang CH, Chen HC, Lin JW, Kuo CW, Shau WY, Lai MS. Risk of hospitalization for upper gastrointestinal adverse events associated with nonsteroidal anti-inflammatory drugs: a nationwide case-crossover study in Taiwan. *Pharmacoepidemiology and drug safety.* Jul 2011;20(7):763-771.

15. Chang CH, Lin JW, Chen HC, Kuo CW, Shau WY, Lai MS. Non-steroidal anti-inflammatory drugs and risk of lower gastrointestinal adverse events: a nationwide study in Taiwan. *Gut.* Oct 2011;60(10):1372-1378.

16. Chang CH, Shau WY, Kuo CW, Chen ST, Lai MS. Increased risk of stroke associated with nonsteroidal anti-inflammatory drugs: a nationwide case-crossover study. *Stroke; a journal of cerebral circulation.* Sep 2010;41(9):1884-1890.

17. Shau WY, Chen HC, Chen ST, et al. Risk of new acute myocardial infarction hospitalization associated with use of oral and parenteral non-steroidal anti-inflammation drugs (NSAIDs): a case-crossover study of Taiwan's National Health Insurance claims database and review of current evidence. *BMC cardiovascular disorders.* 2012;12:4.

18. Discombe G. Agranulocytosis caused by amidopyrine; an avoidable cause of death. *British medical journal.* Jun 14 1952;1(4771):1270-1273.

19. Huguley CM, Jr. AGRANULOCYTOSIS INDUCED BY DIPYRONE, A HAZARDOUS ANTIPYRETIC AND ANALGESIC. *Jama.* Sep 21 1964;189:938-941.

20. Ibanez L, Vidal X, Ballarin E, Laporte JR. Agranulocytosis associated with dipyrone (metamizol). *European journal of clinical pharmacology.* Jan 2005;60(11):821-829.

21. Blaser LS, Tramonti A, Egger P, Haschke M, Krahenbuhl S, Ratz Bravo AE. Hematological safety of metamizole: retrospective analysis of WHO and Swiss spontaneous safety reports. *European journal of clinical pharmacology.* Feb 2015;71(2):209-217.

22. Basak GW, Drozd-Sokolowska J, Wiktor-Jedrzejczak W. Update on the incidence of metamizole sodium-induced blood dyscrasias in Poland. *The Journal of international medical research.* Jul-Aug 2010;38(4):1374-1380.

23. Hamerschlak N, Maluf E, Pasquini R, et al. Incidence of aplastic anemia and agranulocytosis in Latin America--the LATIN study. *Sao Paulo medical journal = Revista paulista de medicina.* May 2 2005;123(3):101-104.

24. Andrade SE, Martinez C, Walker AM. Comparative safety evaluation of non-narcotic analgesics. *Journal of clinical epidemiology.* Dec 1998;51(12):1357-1365.

25. Chaparro LE, Lezcano W, Alvarez HD, Joaqui W. Analgesic effectiveness of dipyrone (metamizol) for postoperative pain after herniorrhaphy: a randomized, double-blind, dose-response study. *Pain practice : the official journal of World Institute of Pain.* Feb 2012;12(2):142-147.

26. Grundmann U, Wornle C, Biedler A, Kreuer S, Wrobel M, Wilhelm W. The efficacy of the non-opioid analgesics parecoxib, paracetamol and metamizol for postoperative pain relief after lumbar microdiscectomy. *Anesthesia and analgesia.* Jul 2006;103(1):217-222, table of contents.

27. Soltesz S, Gerbershagen MU, Pantke B, Eichler F, Molter G. Parecoxib versus dipyrone (metamizole) for postoperative pain relief after hysterectomy : a prospective, single-centre, randomized, double-blind trial. *Clinical drug investigation.* 2008;28(7):421-428.

28. Sener M, Yilmazer C, Yilmaz I, et al. Efficacy of lornoxicam for acute postoperative pain relief after septoplasty: a comparison with diclofenac, ketoprofen, and dipyrone. *Journal of clinical anesthesia.* Mar 2008;20(2):103-108.

29. Sener M, Yilmazer C, Yilmaz I, Caliskan E, Donmez A, Arslan G. Patient-controlled analgesia with lornoxicam vs. dipyrone for acute postoperative pain relief after septorhinoplasty: a prospective, randomized, double-blind, placebo-controlled study. *European journal of anaesthesiology.* Mar 2008;25(3):177-182.

30. Korkmaz Dilmen O, Tunali Y, Cakmakkaya OS, et al. Efficacy of intravenous paracetamol, metamizol and lornoxicam on postoperative pain and morphine consumption after lumbar disc surgery. *European journal of anaesthesiology.* May 2010;27(5):428-432.

31. Brodner G, Gogarten W, Van Aken H, et al. Efficacy of intravenous paracetamol compared to dipyrone and parecoxib for postoperative pain management after minor-to-intermediate surgery: a randomised, double-blind trial. *European journal of anaesthesiology.* Feb 2011;28(2):125-132.

32. Rawal N, Allvin R, Amilon A, Ohlsson T, Hallen J. Postoperative analgesia at home after ambulatory hand surgery: a controlled comparison of tramadol, metamizol, and paracetamol. *Anesthesia and analgesia.* Feb 2001;92(2):347-351.

33. Chauvin M. State of the art of pain treatment following ambulatory surgery. *European journal of anaesthesiology. Supplement.* 2003;28:3-6.

34. Rawal N. Postoperative pain treatment for ambulatory surgery. *Best practice & research. Clinical anaesthesiology.* Mar 2007;21(1):129-148.

35. Warren-Stomberg M, Brattwall M, Jakobsson JG. Non-opioid analgesics for pain management following ambulatory surgery: a review. *Minerva anestesiologica.* Sep 2013;79(9):1077-1087.

36. Ong CK, Seymour RA, Lirk P, Merry AF. Combining paracetamol (acetaminophen) with nonsteroidal antiinflammatory drugs: a qualitative systematic review of analgesic efficacy for acute postoperative pain. *Anesthesia and analgesia.* Apr 1 2010;110(4):1170-1179.

37. Derry CJ, Derry S, Moore RA. Single dose oral ibuprofen plus paracetamol (acetaminophen) for acute postoperative pain. *The Cochrane database of systematic reviews.* 2013;6:Cd010210.

38. Kleinbeck SV. Self-reported at-home postoperative recovery. *Research in nursing & health.* Dec 2000;23(6):461-472.

39. Peters ML, Sommer M, de Rijke JM, et al. Somatic and psychologic predictors of long-term unfavorable outcome after surgical intervention. *Annals of surgery.* Mar 2007;245(3):487-494.

40. Van Agt HM, Essink-Bot ML, Krabbe PF, Bonsel GJ. Test-retest reliability of health state valuations collected with the EuroQol questionnaire. *Social science & medicine.* Dec 1994;39(11):1537-1544.

41. Jakobsson J. Assessing recovery after ambulatory anaesthesia, measures of resumption of activities of daily living. *Current opinion in anaesthesiology.* Dec 2011;24(6):601-604.

42. Wong J, Tong D, De Silva Y, Abrishami A, Chung F. Development of the functional recovery index for ambulatory surgery and anesthesia. *Anesthesiology.* Mar 2009;110(3):596-602.

43. Gramke HF, de Rijke JM, van Kleef M, et al. The prevalence of postoperative pain in a cross-sectional group of patients after day-case surgery in a university hospital. *The Clinical journal of pain.* Jul-Aug 2007;23(6):543-548.

44. Stessel, B.;Fiddelers, A.; Joosten, E.; Hoofwijk, D.; Gramke, H-F.; Buhre, W. Prevalence and predictors of quality of recovery at home after day surgery. *Medicine.* 2015.

45. Maj S, Lis Y. The incidence of metamizole sodium-induced agranulocytosis in Poland. *The Journal of international medical research.* Sep-Oct 2002;30(5):488-495.
