# Supplementary material for: One-month recovery profile and prevalence and predictors of quality of recovery after painful day case surgery: Secondary analysis of a randomized controlled trial
Source: PLoS One. 2021 Jan 26;16(1):e0245774. doi: 10.1371/journal.pone.0245774 (PMC7837485; doi:10.1371/journal.pone.0245774)
Supplement: S2 Protocol — (DOCX) [file pone.0245774.s003.docx]

Metamizole versus NSAID at home after ambulatory surgery: a double-blind randomized controlled trial

Dr. B Stessel

JESSA Ziekenhuis Hasselt

Stadsomvaart 11

3500 Hasselt

Tel.: 0032/479292433

e-mail: bjornstessel@hotmail.com

[1. INTRODUCTION AND RATIONALE 4](#_Toc22731354)

[2. ENDPOINTS 8](#_Toc22731355)

[3. STUDY POPULATION 9](#_Toc22731356)

[3.1 Population 9](#_Toc22731357)

[3.2 Inclusion criteria 9](#_Toc22731358)

[3.3 Exclusion criteria 10](#_Toc22731359)

[3.4 Stopcriteria 11](#_Toc22731360)

[3.5 Sample size calculation 11](#_Toc22731361)

[4. TREATMENT 13](#_Toc22731362)

[4.1 Investigational product 13](#_Toc22731363)

[4.2 Rescue medicatie 13](#_Toc22731364)

[5. STUDY MEDICATION 14](#_Toc22731365)

[5.1 Name and description 14](#_Toc22731366)

[5.2 Product 14](#_Toc22731367)

[5.3 Risks 14](#_Toc22731368)

[5.4 Application 16](#_Toc22731369)

[5.5 Dosage 16](#_Toc22731370)

[5.6 Preparation and labelling of study medication 16](#_Toc22731371)

[5.7 Drug accountability 16](#_Toc22731372)

[6. METHODS 16](#_Toc22731373)

[6.1 Outcome measures 16](#_Toc22731374)

[6.1.1 Primary endpoints 16](#_Toc22731375)

[6.1.2 Secondary endpoints. 17](#_Toc22731376)

[6.2 Randomization and blinding 18](#_Toc22731377)

[6.3 Study procedures 18](#_Toc22731378)

[7. SAFETY 21](#_Toc22731380)

[8. STATISTICAL ANALYSIS 21](#_Toc22731381)

[9. ETHICAL CONSIDERATIONS 23](#_Toc22731382)

[9.1 Legal background 23](#_Toc22731383)

[9.2 Recruitment and informed consent 23](#_Toc22731384)

[9.3 Insurance 24](#_Toc22731385)

[9.4 Reimbursement 24](#_Toc22731386)

[10. PUBLICATION 25](#_Toc22731387)

[10.1 Data management 25](#_Toc22731388)

[10.2 Progress report 25](#_Toc22731389)

[10.3 End report 25](#_Toc22731390)

[10.4 Publication of the results 26](#_Toc22731391)

11. REFERENCES 27

LIST OF ABBREVIATIONS

| **NRS** | **Numeric Rating Scale** |
| --- | --- |
| **MIA** | **Metamizole-induced agranulocytosis** |
| **GSR**  **FRI**  **NSAID**  **EQ-5D**  **SFQ** | **Global Surgical Recovery index**  **Functional Recovery Index**  **Non-Steroidal Anti-Inflammatory Drugs**  **Euro Quality Of Life – 5 Dimensions**  **Surgical Fear Questionnaire** |

# INTRODUCTION AND RATIONALE

Day surgery has been expanding substantially in the past decade, primarily because it is associated with lower costs and it is believed to be as safe as surgery in the inpatient setting. Moreover, it seems that early discharge can contribute to faster recovery and a decreased incidence of hospital associated complications.

In view of the relative absence of major complications, postoperative pain and, in a more extended perspective, quality of recovery (QOR) should be considered the principal endpoints after day surgery ^1-3^. Pain and QOR are two related phenomena encompassing many dimensions in physical, psychological and social health ^4^.

Particularly in the ambulatory setting, good postoperative analgesia is challenging because patients have to control pain at home by themselves ^5^ and the types of analgesics (i.e. no strong opioids) as well as the route of administration (i.e. no epidural, intravenous, subcutaneous or intramuscular route) is limited compared to the inpatient setting. Despite increased awareness and improvements in postoperative pain management over the last decades, the prevalence of outpatients suffering moderate to severe acute postoperative pain at home still remains high and varies from 9-40% ^6-10^. More specific, patients undergoing haemorrhoid surgery, arthroscopic shoulder and knee surgery, and inguinal hernia repair seem to be at highest risk to develop moderate to severe pain and to be poorly recovered on the fourth postoperative day ^3,8^.

Another major disadvantage of the ambulatory setting is related to the absence of postoperative surveillance by professionals. The latter implicates that the individual patient has to assess, without any support, if his/her QOR is normal or not. Unfortunately, there is rather limited information on procedure-specific QOR after day surgery in a more protracted perspective ^3,4,11^. It is of major importance to study QOR profiles after different types of day surgery. This knowledge would allow to discriminate between a normal and pathological health trajectory. Furthermore, the role and effect of pain therapy on QOR profile should be more closely investigated.

Nowadays a multimodal approach to control pain has been advocated in the ambulatory setting. This approach is based on a combination of paracetamol, non-steroidal anti-inflammatory drugs, weak opioids, and local and regional anaesthesia has been advocated in the ambulatory setting ^7,12,13^. Furthermore a systematic meta-analysis has shown that a combination of paracetamol and an NSAID may offer superior analgesia compared to either drug alone ^14^. Consequently, Ibuprofen, a non-steroidal anti-inflammatory drug (NSAID) with a favourable analgesic profile ^15^ in combination with paracetamol comprise our standard multimodal pain treatment model for patients at home after painful day surgery. Nevertheless, NSAID´s are not always sufficiently effective ^8^, have numerous contraindications ^16-20^, and as a result of this are not suitable in up to 25% of all patients ^21^.

Metamizole or dipyrone is a non-opioid compound with potent analgesic, antipyretic and spasmolytic effects ^22^. The analgesic efficacy of intravenous or intramuscular metamizole for pain relief after inpatient surgery is well described ^23-29^. The analgesic efficacy of individual tramadol, metamizole and paracetamol for postoperative analgesia at home after ambulatory hand surgery has also been analyzed ^30^. However, the analgesic efficacy of a combination of paracetamol and metamizole for pain relief at home after day surgery has never been studied.

Hence, in the present study we aim to assess if a combination of metamizole and paracetamol is non-inferior to a combination of the NSAID ibuprofen and paracetamol in the treatment of acute postoperative pain at home after painful day-case surgery. We hypothesize that ambulatory patients postoperatively treated with paracetamol/metamizole will achieve equal or even better pain relief compared to patients treated with paracetamol/ibuprofen. In addition, we aim to assess and compare QOR profiles of both groups. We hypothesize that each type of day surgery included in our trial will have a unique QOR profile, significantly different from the QOR profile of other types of day surgery.

The primary goal of this study consists of two parts:

1. The evaluation of the analgesic effect of the combination of paracetamol and metamizole compared to the standard pain protocol (paracetamol/ibuprofen) in patients undergoing painful ambulant surgery. Our hypothesis is that the analgesic effect of a combination of paracetamol and metamizole is equal compared to the standard pain protocol.
2. The evaluation of the recovery profile of the two treatment groups and of the different types of ambulant surgery. Het bestuderen en vergelijken van het herstelprofiel van de twee behandelgroepen en van verschillende types van pijnlijke ambulante chirurgie.

Secondary endpoints are the evaluation of potential sides effects of the study medication, the use of rescue-mediation (dipidolor iv in the PACU and tramadol oral at home), satisfaction of the patients, compliance and identification of potential predictors of postoperative pain and recovery.

**Hypotheses**

1. A combination of metamizole and paracetamol can reduce postoperative pain compared to a combination of ibuprofen and paracetamol.
2. Each type of ambulant surgery has a unique recovery profile, significant different from the recovery profiles of other ambulant surgery
3. A combination of metamizole and paracetamol results in a better recovery after ambulant surgery compared to a combination of ibuprofen and paracetamol.
4. There are few (or none) side effects observed using oral metamizole during postoperative treatment after ambulant surgery.
5. Satisfaction of the patients is high in both groups.

# ENDPOINTS

**Primary endopoints**

- Postoperative pain measured by NRS.

Research question: Is there a difference in postoperative pain intensity between the different treatment groups? A difference of more than 1 point (NRS) will be considered clinical relevant. This small difference is chosen due to the non-inferiority character of the study.

- Postoperative recovery measured by GSR and the differences of EQ-5D and FRI.

Research question: Is there a difference in the recovery of patients in the different treatment groups and in the difference types of ambulant surgery?

**Secondary endpoints**

- Side effects of the used analgetica: pyrosis, signs of agrunlocytosis or thrombocytopenia
- Satisfaction of the patient
- Use of rescue medication (dipidolor in PACU and tramadol at home)
- Compliance
- Predictors of postoperative pain and recovery STUDY DESIGN

This study is a mono center, double blind, prospective, randomized non-inferiority trial with 2 treatment groups.

Group 1: Postoperative days 1, 2, 3, 4: paracetamol 1000mg 4x/day, ibuprofen 600mg 3x/ day (standard JESSA hospital)

Group 2: Postoperative days 1, 2, 3, 4: paracetamol 1000mg 4x/day, metamizole 1000mg 3x/day

**Timeframe:**

The study will last 6 months dependent on the number of suitable patients, this can take up to 8 months.

# STUDY POPULATION

## Population

Consecutive adult patients undergoing elective hemorrhoid surgery, arthroscopic shoulder or knee surgery, or inguinal hernia repair in a day care setting will be informed about the study on their preoperative visit by the surgeon and they will be provided with a patient information sheet.

## Inclusion criteria

- Patients between 18 and 70 years old
- ASA classification 1,2 of 3
- Patients undergoing one of the following surgeries: Hemorrhoids, knee or shoulder arthroscopy or inguinal hernia repair
- Weight > 50kg and < 100kg

## Exclusion criteria

- Age <18 years and >70 years
- Unability to score pain with the NRS, for instance dementia etc
- Language barrier
- Preoperative opioid therapy
- Fibromyalgy, CRPS, chronic pain
- Sensitivity to metamizole, alcohol, paracetamol or ibuprofen (and other NSAIDs)
- Porphyria
- Congenital deficiency of glucose-6-fosfate dehydrogenase
- Pregnancy or lactation
- Kidney or liver disfunction
- Asthma
- Rhinosinusitis or nasal polyps
- Severe COPD, emphysema
- Chronic constipation
- Use of anti-rheumatic medication
- Haematological disease
- Hypotension
- Ulcus pepticum or internal bleeding: active or anamneses
- Gastrointestinal bleeding or perforation due to the use of cyclooxygenase-inhibitors in anamneses
- Heartfailure
- Opioid abusus in anamneses
- Chronic use of alcohol
- Use of central antidepressants such as benzodiazepines
- Fever or other signs of infection
- Only in patients undergoing shoulder arthroscopy: refusal of an interscalenus block

## Stopcriteria

- - Decision of the patient
  - Surgical complications requiring a revision or stay in the hospital

## Sample size calculation

The primary outcome measures are the average postoperative pain intensity measured by an 11-point Numeric Rating Scale (NRS) on the first postoperative day as well as the QOR profile. As QOR profile is strongly influenced by postoperative pain intensity, sample size calculation will be based on expected postoperative pain intensity. Based on previous studies, we assume a standard deviation of the NRS-scores of 2,5 on the first postoperative day and 1,7 on the fourth postoperative day 3,5. A difference in mean average NRS-score of 1 point or less is considered non-inferior. Therefore, the present study must have the power to detect a difference of more than 1 point to reject the null-hypothesis that the analgesic power of a combination of metamizole and paracetamol is inferior compared to a combination of ibuprofen and paracetamol. Based on these assumptions, we will require 78 patients in each group to have a power of at least 80 % (β = 0.2). To determine non-inferiority, we will compute 95% confidence intervals for the difference in primary endpoints. The sample size will be inflated to 100 participants per group (200 in total) to account for a possible 22 % loss-to-follow-up.

# TREATMENT

## Investigational product

Postoperative analgesia of metamizole in combination with paracetamol.

## Rescue medicatie

Rescuemedication in both groups: Tramadol odis 50mg

# STUDY MEDICATION

## Name and description

Metamizole:

metamizole is an analgeticum with a unique mechanism.

Paracetamol:

Paracetamol is a non-opioïd analgeticum.

Ibuprofen:

Ibuprofen belongs to the non-steroid anti-inflammatory drugs (NSAIDs)

## Product

See SmPc of all products.

## Risks

Paracetamol:

Paracetamol is part of the standard pain protocol for postoperative analgesia in Jessa Hospital. The risk for the patient is not elevated by participation in the study. In therapeutical doses as used in this study, little side effects occur. Sensibility reaction such as urticaria can occur as well as fever, thrombocytopenia and hemolytic anemia although this is rare. Serious side effects and complications can occur after toxic dosage and after persistent use for a longer time frame. Here, paracetamol will only be used during 96 hours.

Ibuprofen:

Ibuprofen is part of the standard pain protocol for postoperative analgesia in Jessa Hospital, contra indications taking into account. The risk for the patient is not elevated by participation in the study. Again, ibuprofen will only be used in a short time period of 96 hours. Sensibility reactions such as urticaria or pruritus are possible. Gastro-intestinal side effects are relatively frequent, namely reflux, abdominal pain, nausea and constipation. Severe gastro-intestinal side effects such as bleeding in the stomach or bowel, ulcus pepticum, perforation of stomach or bowel are less frequent and are correlated with a longer time frame. Furthermore, ibuprofen has an inhibitory effect on the aggregation of thrombocytes which can cause a longer bleeding time. Disfunction of the kidneys, lung edema and heart failure are possible.

Metamizole

Metamizole is not a part of the standard pain protocol for postoperative analgesia in Jessa Hospital. During this study, metamizole will only be used in a short time frame (96 hours). Sensibility reactions such as allergic skin reactions (erythema, urticaria, pruritus), bronchospasm and, although very rare, an anaphylactic shock can occur. Furthermore, discomfort in the stomach and bowel are possible. Metamizole will give urine a red color which is not harmful. In isolated cases and in case of toxic dosage reversible disfunction in the kidneys is possible. In rare cases, agranulocytosis can occur which manifests in sepsis, angina and benign ulcers in the mouth. Exceptionally thrombocytopenia can occur with elevated risk of bleeding of the skin. Hypotension can occur occasionally when iv applied, not related to an allergic reaction.

## Application

All study medication will be provided in tablet, for oral use.

## Dosage

The chosen dosage of metamizole is according to literature. The chosen dosages of paracetamol and ibuprofen are the same as the dosages used at the ambulant center in the Jessa hospital.

## Preparation and labelling of study medication

Standard package for paracetamol. Blinded package performed by the hospital pharmacy for ibuprofen and metamizole.

## Drug accountability

The study medication will be delivered by the hospital pharmacy in blisters. Each box will contain the study medication needed during the study period (96 hours).

# METHODS

## Outcome measures

### Primary endpoints

Primary endpoints are:

1. Average postoperative pain intensity measured by an 11-point Numeric Rating Scale (NRS; where 0 = no pain, and 10 = worst pain imaginable) at postoperative day 1.
2. QOR profile measured by the Functional Recovery Index (FRI), the 1-item Global Surgical Recovery (GSR) index and the EuroQol (EQ-5D) questionnaire at days 1, 2, 3, 4, 7, 14 and 28 postoperatively.
   - The convenient and also validated GSR index represents a single question about the extent to which patients considered themselves to be recovered from the surgery (0 – 100%).
   - The FRI is a questionnaire-based instrument specifically developed to assess post-discharge functional QOR after day surgery and covers 14 items grouped under 3 factors (pain and social activity, lower limb activity and general physical activity).
   - The 5-dimensional EQ-5D is a non-disease specific instrument developed for describing and valuing health-related quality of life 32 and has already been used to assess intermediate (4 days) and late (2 weeks – 6 months) quality of recovery after day surgery.

### Secondary endpoints.

The following outcomes will also be assessed:

- Postoperative pain intensity at movement and rest measured by an 11-point Numeric Rating Scale (NRS; where 0 = no pain, and 10 = worst pain imaginable) at discharge and day 1, 2, 3, 4, 7, 14 and 28 postoperatively.
- Average postoperative pain intensity measured by an 11-point Numeric Rating Scale (NRS; where 0 = no pain, and 10 = worst pain imaginable) at day 0, 2, 3, 4, 7, 14 and 28 postoperatively.
- Adherence to study medication at day 1, 2, 3 and 4 postoperatively.
  - Definition compliance: Full compliance: analgesia use as prescribed “Yes”, No compliance: analgesia use as prescribed “No”.
- Adverse effects of study medication (i.e. pyrosis, signs of agranulocytosis or thrombocytopenia)
- Total postoperative intravenous piritramide consumption at the PACU (milligram)
- Use of rescue medication (tramadol at home) at day 1, 2, 3 and 4 postoperatively (yes/no).
- Satisfaction with study medication, surgery and hospital care and telephone follow-up measured at the seventh postoperative day by an 11-point Numeric Rating Scale (NRS).

## Randomization and blinding

Participants will be randomly assigned to the MP or the IP group using a computer-generated random allocation sequence, created by the study statistician. Randomization will be stratified for type of surgery. Each patient will receive a unique randomized test number corresponding to the specified drug, according to the group allocation. The randomisation list remains with the study statistician and the hospital pharmacy for the whole duration of the study. Hence, the patients participating in the trial, the treating physicians, the researchers dispensing the medication and assessing outcomes (i.e. 4 trained resident physicians and 1 study nurse) and the data-managers will be blinded for group allocation.

## Study procedures

**Preoperative procedure:**

After obtaining written informed consent, the researcher will also record participants’: age, gender, Body Mass Index (BMI), ASA classification, work status, highest level of education, fear of the surgical procedure (using an 8-item surgical fear questionnaire), pre-operative pain (the baseline NRS-score), expected pain (NRS-score), baseline GSR, FRI and EQ-5D, and the history of (related) surgery. Hereafter, patients will be randomized in a 1:1 ratio to either of the two study groups: a combination of metamizole and paracetamol (MP) or a combination of ibuprofen and paracetamol (IP) group. Patients in the MP-group (experimental arm) will be instructed to take metamizole 1gr orally three times a day for four days and patients in the IP-group (control arm) will be instructed to take ibuprofen 600mg orally three times a day for four days. All patients will also be treated with paracetamol 1gr orally four times a day during the entire study period. The first dose of study medication (metamizole and paracetamol (MP) or ibuprofen and paracetamol (IP) will be given 30 minutes before surgery. Rescue medication consists of tramadol 50mg orally and will be taken up to three times a day only if pain relief wasn´t satisfactory with study medication. Patients will be instructed to take their trial medication as prescribed and will be provided with a detailed medication schedule. Furthermore, they will be called by telephone daily and asked if they took their trial medication as prescribed.

**Perioperative procedure:**

All patients scheduled for an arthroscopic shoulder procedure will receive an interscalene block preoperatively.

In addition, general anesthesia will be induced with alfentanil 10mcg/kg IV, sufentanil 0,15mcg/kg IV and propofol 2mg/kg IV. Patients undergoing arthroscopic shoulder surgery or laparoscopic inguinal hernia repair will also receive rocuronium 20-40mg before endotracheal intubation. A laryngeal mask airway will be inserted in all other patients. Anesthesia will be maintained with sevoflurane in a mixture of 50:50 air/oxygen. Before the end of surgery, all patients will receive ondansetron 4mg IV. Furthermore, wound infiltration with local anesthesia (bupivacaine 0,5%) will be performed in all patients except those receiving an interscalene block. Duration of surgery will be recorded.

**Postoperative procedure:**

Postoperatively, all patients will be treated with subsequent bolus injections of piritramide 2mg intravenously until an NRS ≤ 3 is reached/achieved in the Post Anesthesia Care Unit (PACU). Before hospital discharge, patients will receive the study medication and instructions for use. Dropout criteria are surgical complications leading to either revision surgery or unanticipated hospital admission.

All patients will receive reminders by phone call on day 1-4, day 7, day 14 and day 28 after the surgery to reduce a loss to follow-up. All data will be asked over the phone.

Safety:

All possible adverse effects of study medication will be explained thoroughly to all patients who meet the eligibility criteria. All participants will be questioned about adverse events at each telephone call (day 1, 2, 3, 4, 7, 14 and 28 postoperatively). Patients will be specifically asked whether they experienced postoperative nausea and vomiting, pyrosis or stomachache, obstipation, anaphylaxis, fever, chills, mouth ulcers, a sore throat or signs of infection, petechiae and bleeding diathesis. Furthermore, patients are instructed to contact immediately a research assistant by phone if they experience moderate to severe signs of infection and/or bleeding diathesis. A complete blood count will then be performed to exclude leukopenia, anemia and thrombocytopenia and trial medication will be withdrawn from the patient. Such an event will be reported as a serious adverse event (SAE).

# SAFETY

All SAEs will be reported to the ethical committee which approved the study according to the Declaration of Helsinki.

# STATISTICAL ANALYSIS

Participants’ data will be recorded in individual participant record booklets. Coded, depersonalized data will be entered into a web-based questionnaire (Questback) and then exported to SPSS version 21. Database access will be restricted to the authorised research team. Participants’ study information will not be released outside of the study without the written permission of the participant.

All primary and secondary endpoints will be analyzed on a per-protocol basis according to a non-inferiority design. As a sensitivity analysis, we will compare these results to an intention to treat analysis. Data will be presented as mean values +/- SD, numbers (n), and percentages (%). P values < 0.05 will be considered statistically significant. Missing baseline values will be imputed using multiple imputation. The number of imputations will be set to 10. To determine non-inferiority for the difference in NRS score on the first day after surgery, we will compute 95% confidence intervals. The QOR profiles will be assessed using linear mixed models, taking time, type of surgery and group assignment into account. Differences between the groups on secondary outcomes will be analyzed using the Student t test for continuous outcomes, except for the pain scores, since these are often heavily skewed. For those outcomes, we will use the Mann- Whitney U test. All categorical variables will be compared using Pearson’s χ2 test. For multivariate analysis the following potential confounders will be assessed: surgical fear, preoperative pain (baseline NRS), expected pain, baseline FRI and EQ-5D, age, sex, work status, educational level, ASA classification, BMI, history of related surgery, type of surgery, and duration of surgery. In addition, changes in the QOR scores over time in the postoperative period will be analyzed using linear mixed models and we will use logistic regression to explore possible predictors for QOR. All analyses will be performed using SPSS version 21.

# ETHICAL CONSIDERATIONS

## Legal background

The study will be conducted according to:

**“WORLD MEDICAL ASSOCIATION DECLARATION OF HELSINKI**

**Ethical Principles for Medical Research Involving Human Subjects**

Adopted by the 18th WMA General Assembly, Helsinki, Finland, June 1964, and amended by

the

29th WMA General Assembly, Tokyo, Japan, October 1975

35th WMA General Assembly, Venice, Italy, October 1983

41st WMA General Assembly, Hong Kong, September 1989

48th WMA General Assembly, Somerset West, Republic of South Africa, October 1996

and the 52nd WMA General Assembly, Edinburgh, Scotland, October 2000

Note of Clarification on Paragraph 29 added by the WMA General Assembly, Washington 2002

Note of Clarification on Paragraph 30 added by the WMA General Assembly, Tokyo 2004”

## Recruitment and informed consent

At least 24 hours before surgery patients will be informed about the study oral and written by a member of the research team or a study nurse. These information will be given at the preoperative consultation. Patients are given sufficient time to reflect and ask questions until the day of surgery. By signing an informed consent, patients

agree to participate in the study. This is possible until the day of surgery. Participation is volunteerly and the patient has the right to change his opinion at any time point during the study. The quality of care will not be affected. Door verlenen de patiënten hun toestemming voor de inclusie in the studie. All records will be coded with only acces of the researchteam.

## Insurance

The investigators have an insurance polis in case of any injury or damage of participants of the study.

## Reimbursement

None

# PUBLICATION

## Data management

Participants’ data will be recorded in individual participant record booklets. Coded, depersonalized data will be entered into a web-based questionnaire (Questback) and then exported to SPSS version 21. Database access will be restricted to the authorised research team. Participants’ study information will not be released outside of the study without the written permission of the participant. Hard copies of participants’ data and analyses will be kept in storage for 15 years.

## Progress report

The principal investigator delivers a progress report once a year to the ethical committee. This report includes information concerning date of inclusion of the first patient, the number of recruited participants, drop-outs, SAEs etcetera.

## End report

The principal investigator notifies the ethical committee and the government (FAGG) at the end of the study (within 90 days). The end of the study is defined as the last consult of the last patient over the phone at day 28 after surgery.
The principal investigator will notify the ethical committee and FAGG in case of early discontinuation of the trial and the cause of the early discontinuation within 15 days.
An end report will be presented to the ethical committee and FAGG within one year after the end of the study, with all publications and abstracts included.

## Publication of the results

The results of this study will be published in a “peer-reviewed” scientific journal. The institute and/or investigator have the right to publish the results of this study and copyright of these publications will be owned by the investigator.

**11. REFERENCES**

1. Wong J, Tong D, De Silva Y, Abrishami A, Chung F. Development of the functional recovery index for ambulatory surgery and anesthesia. *Anesthesiology.* Mar 2009;110(3):596-602.

2. Jakobsson J. Assessing recovery after ambulatory anaesthesia, measures of resumption of activities of daily living. *Current opinion in anaesthesiology.* Dec 2011;24(6):601-604.

3. Stessel B, Fiddelers AA, Joosten EA, Hoofwijk DM, Gramke HF, Buhre WF. Prevalence and Predictors of Quality of Recovery at Home After Day Surgery. *Medicine (Baltimore).* Sep 2015;94(39):e1553.

4. Tran TT, Kaneva P, Mayo NE, Fried GM, Feldman LS. Short-stay surgery: what really happens after discharge? *Surgery.* Jul 2014;156(1):20-27.

5. Gramke HF, de Rijke JM, van Kleef M, et al. Predictive factors of postoperative pain after day-case surgery. *The Clinical journal of pain.* Jul-Aug 2009;25(6):455-460.

6. Wu CL, Berenholtz SM, Pronovost PJ, Fleisher LA. Systematic review and analysis of postdischarge symptoms after outpatient surgery. *Anesthesiology.* Apr 2002;96(4):994-1003.

7. Rawal N. Postoperative pain treatment for ambulatory surgery. *Best practice & research. Clinical anaesthesiology.* Mar 2007;21(1):129-148.

8. Gramke HF, de Rijke JM, van Kleef M, et al. The prevalence of postoperative pain in a cross-sectional group of patients after day-case surgery in a university hospital. *The Clinical journal of pain.* Jul-Aug 2007;23(6):543-548.

9. McGrath B, Elgendy H, Chung F, Kamming D, Curti B, King S. Thirty percent of patients have moderate to severe pain 24 hr after ambulatory surgery: a survey of 5,703 patients. *Canadian journal of anaesthesia = Journal canadien d'anesthesie.* Nov 2004;51(9):886-891.

10. Beauregard L, Pomp A, Choiniere M. Severity and impact of pain after day-surgery. *Canadian journal of anaesthesia = Journal canadien d'anesthesie.* Apr 1998;45(4):304-311.

11. Brattwall M, Warren Stomberg M, Rawal N, Segerdahl M, Jakobsson J, Houltz E. Patients' assessment of 4-week recovery after ambulatory surgery. *Acta anaesthesiologica Scandinavica.* Jan 2011;55(1):92-98.

12. Warren-Stomberg M, Brattwall M, Jakobsson JG. Non-opioid analgesics for pain management following ambulatory surgery: a review. *Minerva anestesiologica.* Sep 2013;79(9):1077-1087.

13. Chauvin M. State of the art of pain treatment following ambulatory surgery. *European journal of anaesthesiology. Supplement.* 2003;28:3-6.

14. Ong CK, Seymour RA, Lirk P, Merry AF. Combining paracetamol (acetaminophen) with nonsteroidal antiinflammatory drugs: a qualitative systematic review of analgesic efficacy for acute postoperative pain. *Anesthesia and analgesia.* Apr 1 2010;110(4):1170-1179.

15. Derry CJ, Derry S, Moore RA. Single dose oral ibuprofen plus paracetamol (acetaminophen) for acute postoperative pain. *The Cochrane database of systematic reviews.* 2013;6:Cd010210.

16. Castellsague J, Riera-Guardia N, Calingaert B, et al. Individual NSAIDs and upper gastrointestinal complications: a systematic review and meta-analysis of observational studies (the SOS project). *Drug safety.* Dec 1 2012;35(12):1127-1146.

17. Chang CH, Chen HC, Lin JW, Kuo CW, Shau WY, Lai MS. Risk of hospitalization for upper gastrointestinal adverse events associated with nonsteroidal anti-inflammatory drugs: a nationwide case-crossover study in Taiwan. *Pharmacoepidemiology and drug safety.* Jul 2011;20(7):763-771.

18. Chang CH, Lin JW, Chen HC, Kuo CW, Shau WY, Lai MS. Non-steroidal anti-inflammatory drugs and risk of lower gastrointestinal adverse events: a nationwide study in Taiwan. *Gut.* Oct 2011;60(10):1372-1378.

19. Chang CH, Shau WY, Kuo CW, Chen ST, Lai MS. Increased risk of stroke associated with nonsteroidal anti-inflammatory drugs: a nationwide case-crossover study. *Stroke; a journal of cerebral circulation.* Sep 2010;41(9):1884-1890.

20. Shau WY, Chen HC, Chen ST, et al. Risk of new acute myocardial infarction hospitalization associated with use of oral and parenteral non-steroidal anti-inflammation drugs (NSAIDs): a case-crossover study of Taiwan's National Health Insurance claims database and review of current evidence. *BMC cardiovascular disorders.* 2012;12:4.

21. Benhamou D, Bouaziz H, Zerrouk N, Preaux N. Audit of ketoprofen prescribing after orthopedic and general surgery. *Canadian journal of anaesthesia = Journal canadien d'anesthesie.* Feb 1999;46(2):109-113.

22. Huber M, Andersohn F, Sarganas G, et al. Metamizole-induced agranulocytosis revisited: results from the prospective Berlin Case-Control Surveillance Study. *European journal of clinical pharmacology.* Feb 2015;71(2):219-227.

23. Chaparro LE, Lezcano W, Alvarez HD, Joaqui W. Analgesic effectiveness of dipyrone (metamizol) for postoperative pain after herniorrhaphy: a randomized, double-blind, dose-response study. *Pain practice : the official journal of World Institute of Pain.* Feb 2012;12(2):142-147.

24. Grundmann U, Wornle C, Biedler A, Kreuer S, Wrobel M, Wilhelm W. The efficacy of the non-opioid analgesics parecoxib, paracetamol and metamizol for postoperative pain relief after lumbar microdiscectomy. *Anesthesia and analgesia.* Jul 2006;103(1):217-222, table of contents.

25. Soltesz S, Gerbershagen MU, Pantke B, Eichler F, Molter G. Parecoxib versus dipyrone (metamizole) for postoperative pain relief after hysterectomy : a prospective, single-centre, randomized, double-blind trial. *Clinical drug investigation.* 2008;28(7):421-428.

26. Sener M, Yilmazer C, Yilmaz I, et al. Efficacy of lornoxicam for acute postoperative pain relief after septoplasty: a comparison with diclofenac, ketoprofen, and dipyrone. *Journal of clinical anesthesia.* Mar 2008;20(2):103-108.

27. Sener M, Yilmazer C, Yilmaz I, Caliskan E, Donmez A, Arslan G. Patient-controlled analgesia with lornoxicam vs. dipyrone for acute postoperative pain relief after septorhinoplasty: a prospective, randomized, double-blind, placebo-controlled study. *European journal of anaesthesiology.* Mar 2008;25(3):177-182.

28. Korkmaz Dilmen O, Tunali Y, Cakmakkaya OS, et al. Efficacy of intravenous paracetamol, metamizol and lornoxicam on postoperative pain and morphine consumption after lumbar disc surgery. *European journal of anaesthesiology.* May 2010;27(5):428-432.

29. Brodner G, Gogarten W, Van Aken H, et al. Efficacy of intravenous paracetamol compared to dipyrone and parecoxib for postoperative pain management after minor-to-intermediate surgery: a randomised, double-blind trial. *European journal of anaesthesiology.* Feb 2011;28(2):125-132.

30. Rawal N, Allvin R, Amilon A, Ohlsson T, Hallen J. Postoperative analgesia at home after ambulatory hand surgery: a controlled comparison of tramadol, metamizol, and paracetamol. *Anesthesia and analgesia.* Feb 2001;92(2):347-351.
